# Global, regional, and national burden of Parkinson's disease, 1990–2016: a systematic analysis for the Global Burden of Disease Study 2016

**DOI:** 10.1016/S1474-4422(18)30295-3

**Published:** 2018-11

**Authors:** GBD 2016 Parkinson's Disease Collaborators, E. Ray Dorsey, Alexis Elbaz, Emma Nichols, Foad Abd-Allah, Ahmed Abdelalim, Jose C. Adsuar, Mustafa Geleto Ansha, Carol Brayne, Jee-Young J Choi, Daniel Collado-Mateo, Nabila Dahodwala, Huyen Phuc Do, Dumessa Edessa, Matthias Endres, Seyed-Mohammad Fereshtehnejad, Kyle J Foreman, Fortune Gbetoho Gankpe, Rahul Gupta, Graeme J. Hankey, Simon I. Hay, Mohamed I Hegazy, Desalegn T. Hibstu, Amir Kasaeian, Yousef Khader, Ibrahim Khalil, Young-Ho Khang, Yun Jin Kim, Yoshihiro Kokubo, Giancarlo Logroscino, João Massano, Norlinah Mohamed Ibrahim, Mohammed A. Mohammed, Alireza Mohammadi, Maziar Moradi-Lakeh, Mohsen Naghavi, Binh Thanh Nguyen, Yirga Legesse Nirayo, Felix Akpojene Ogbo, Mayowa Ojo Owolabi, David M. Pereira, Maarten J Postma, Mostafa Qorbani, Muhammad Aziz Rahman, Kedir T. Roba, Hosein Safari, Saeid Safiri, Maheswar Satpathy, Monika Sawhney, Azadeh Shafieesabet, Mekonnen Sisay Shiferaw, Mari Smith, Cassandra E I Szoeke, Rafael Tabarés-Seisdedos, Nu Thi Truong, Kingsley Nnanna Ukwaja, Narayanaswamy Venketasubramanian, Santos Villafaina, Kidu gidey weldegwergs, Ronny Westerman, Tissa Wijeratne, Andrea S. Winkler, Bach Tran Xuan, Naohiro Yonemoto, Valery L Feigin, Theo Vos, Christopher J L Murray

## Abstract

**Background:**

Neurological disorders are now the leading source of disability globally, and ageing is increasing the burden of neurodegenerative disorders, including Parkinson's disease. We aimed to determine the global burden of Parkinson's disease between 1990 and 2016 to identify trends and to enable appropriate public health, medical, and scientific responses.

**Methods:**

Through a systematic analysis of epidemiological studies, we estimated global, regional, and country-specific prevalence and years of life lived with disability for Parkinson's disease from 1990 to 2016. We estimated the proportion of mild, moderate, and severe Parkinson's disease on the basis of studies that used the Hoehn and Yahr scale and assigned disability weights to each level. We jointly modelled prevalence and excess mortality risk in a natural history model to derive estimates of deaths due to Parkinson's disease. Death counts were multiplied by values from the Global Burden of Disease study's standard life expectancy to compute years of life lost. Disability-adjusted life-years (DALYs) were computed as the sum of years lived with disability and years of life lost. We also analysed results based on the Socio-demographic Index, a compound measure of income per capita, education, and fertility.

**Findings:**

In 2016, 6·1 million (95% uncertainty interval [UI] 5·0–7·3) individuals had Parkinson's disease globally, compared with 2·5 million (2·0–3·0) in 1990. This increase was not solely due to increasing numbers of older people, because age-standardised prevalence rates increased by 21·7% (95% UI 18·1–25·3) over the same period (compared with an increase of 74·3%, 95% UI 69·2–79·6, for crude prevalence rates). Parkinson's disease caused 3·2 million (95% UI 2·6–4·0) DALYs and 211 296 deaths (95% UI 167 771–265 160) in 2016. The male-to-female ratios of age-standardised prevalence rates were similar in 2016 (1·40, 95% UI 1·36–1·43) and 1990 (1·37, 1·34–1·40). From 1990 to 2016, age-standardised prevalence, DALY rates, and death rates increased for all global burden of disease regions except for southern Latin America, eastern Europe, and Oceania. In addition, age-standardised DALY rates generally increased across the Socio-demographic Index.

**Interpretation:**

Over the past generation, the global burden of Parkinson's disease has more than doubled as a result of increasing numbers of older people, with potential contributions from longer disease duration and environmental factors. Demographic and potentially other factors are poised to increase the future burden of Parkinson's disease substantially.

**Funding:**

Bill & Melinda Gates Foundation.

## Introduction

Neurological disorders are now the leading source of disability globally.^1^ Among neurological disorders examined in the Global Burden of Disease, Injuries, and Risk Factors Study (GBD) 2015, Parkinson's disease was the fastest growing in prevalence, disability, and deaths. In that study,^1^ the overall number of people affected by the disease was estimated to have more than doubled globally from 1990 to 2015. Previous studies have examined the epidemiology of Parkinson's disease for different parts of the world,^2^ including systematic reviews on the prevalence of Parkinson's disease.^3^, ^4^ However, none has examined change in prevalence, disability, and deaths in detail over the past generation for the entire world and across all countries. In GBD 2015, we identified larger variation in Parkinson's disease death rate estimates over time and between countries than we observed in prevalence estimates.^1^ This pattern suggested that coding practices rather than real changes over time and location were responsible, similar to what was observed for dementia.^1^

The prevalence of a disease reflects both the incidence and the duration of disease. The incidence of Parkinson's disease is linked to risk and protective factors.^2^, ^5^, ^6^ The most important risk factor is age, but the risk of Parkinson's disease also appears to be associated with industrial chemicals and pollutants, such as pesticides,^7^ solvents,^7^ and metals.^8^, ^9^ Conversely, smoking is associated with a decreased risk of Parkinson's disease,^10^ but whether this association is causal is debatable.^11^ The factors that affect disease duration are less well known, but increasing longevity also translates into longer disease duration.^3^, ^12^ Therefore, as ageing and industrialisation increase globally and smoking decreases in some regions, the prevalence of Parkinson's disease seems poised to increase.^13^, ^14^ Detailed estimates of the disease burden can help to evaluate the effect of these risk factors and inform efforts to prevent the disease and to care for and treat those affected by the condition.

**Research in context**

**Evidence before this study**

The Global Burden of Diseases, Injuries, and Risk Factors Study (GBD) 2015 examined the epidemiology of Parkinson's disease for different parts of the world and showed that the number of people affected by the condition had more than doubled globally from 1990 to 2015. The increase in deaths from Parkinson's disease was greater than the increase in prevalence, and the large variation in death rates between countries was suggestive of a change in coding practices rather than greater death rates among Parkinson's disease cases. For pragmatic reasons, systematic reviews for Parkinson's disease are scheduled every other iteration of GBD. For GBD 2016, we updated our GBD 2013 PubMed search without language restrictions using the terms (((“Parkinson disease” AND “epidemiology”) AND (“2011/01/01”[PDat]: “2015/12/31”[PDat])) AND (“Parkinson disease” AND “epidemiology”)) to identify articles published between Jan, 1, 2011, and Dec, 31, 2015. Papers were selected if representative of the general population and identification of cases was based on our reference case definition (the presence of at least two of four primary symptoms: rest tremor, bradykinesia, stiffness of limbs and torso, and postural instability) or alternative case definitions (UK Parkinson's Disease Society Brain Bank criteria, and doctor's diagnosis based on International Classification of Diseases codes and prescription of medications specifically for Parkinson's disease).

**Added value of this study**

We used the results of this search to obtain the data needed to estimate global, regional, and country-specific prevalence and years lived with disability for Parkinson's disease from 1990 to 2016. To address the possible measurement error in Parkinson's disease death rates as reported by vital registration systems, we used a method that was previously applied to dementia in GBD 2015. In a natural history modelling approach, we assume a constant risk of death in Parkinson's disease cases over time and between locations and let the death rates be determined by variations in prevalence. Although the assumption of similar mortality risk in all time periods and countries is problematic, it produces less error than the large variation in death rates estimated previously. We also explored variation in the burden by age, sex, country, region, and Socio-demographic Index. This study showed that counts of prevalence, mortality, and disability-adjusted life-years more than doubled from 1990 to 2016, and that this increase was not solely due to increasing numbers of older people because age-standardised rates also increased in most regions. In addition, the burden of Parkinson's disease increased with increasing Socio-demographic Index.

**Implications of all the available evidence**

Neurological disorders are now the leading source of disability in the world, and Parkinson's disease is the fastest growing of these disorders. As the population ages and life expectancy increases, the number of individuals with Parkinson's disease will continue to increase as well as the duration of the disease, leading to more patients with advanced Parkinson's disease. To address this burden, primary prevention strategies based on the underlying causes of Parkinson's disease and more effective treatments than are currently available are required. Additional incidence and prevalence studies are needed, especially in areas in which little data are available, to examine time trends and the factors that drive them.

As part of GBD 2016, we aimed to examine the changes from 1990 to 2016 in counts and age-standardised rates of Parkinson's disease for prevalence, disability, and deaths by location and by the Socio-demographic Index (SDI), a composite measure of income per capita, education, and fertility.^15^

## Methods

### Overview

The general methods for the studies on the global, regional, and national burden of neurological disorders have been published previously,^1^ and key aspects are summarised in the appendix. Additional information on derivation of non-fatal and fatal estimates are provided in the appendix as well as on Global Health Data Exchange.

### Data sources

The International Classification of Diseases ninth revision (ICD-9) codes used in cause of death analyses for Parkinson's disease are 332 (Parkinson's disease), 332.0 (paralysis agitans), and 332.1 (secondary parkinsonism), and the corresponding ICD-10 codes are G20 (Parkinson's disease), G21 (secondary parkinsonism), and G22 (parkinsonism in diseases classified elsewhere). The reference case definition for Parkinson's disease used in many epidemiological studies is the presence of at least two of the four primary symptoms: rest tremor, bradykinesia, stiffness of the limbs and torso, and postural instability. We also accepted alternative definitions, including the UK Parkinson's Disease Society Brain Bank criteria,^16^ in addition to ICD-9 and ICD-10 codes, a doctor's diagnosis of Parkinson's disease, and the prescription of Parkinson's disease-specific medications. We also added 3 years of medical claims data (years 2000, 2010, and 2012) from the USA; for a disease such as Parkinson's disease, the data from claims sources would be expected to match true prevalence under the expectation that most patients would be receiving medical attention each year. If datapoints from epidemiological studies spanned ages of more than 20 years, we split the datapoints using the age pattern from the USA, for which the most detailed age data were available.

### Disease model

For Parkinson's disease, we have seen large inconsistencies between the cause of death data and the non-fatal data. For example, US vital registration data show a greater than three times increase in the age-standardised rates of death from Parkinson's disease since 1980 without a corresponding increase in the prevalence data over the same time period (appendix). Likewise, we found a greater than 25 times difference across different countries in age-standardised mortality rates for the most recent year of vital registration data available at the time of GBD 2016 (see Causes of Death Visualization), and we did not see such heterogeneity between countries in our non-fatal data. Therefore, these differences are probably the result of changes and inconsistencies in coding practices for certifying deaths from Parkinson's disease. To correct for this bias, we jointly modelled the prevalence and mortality from Parkinson's disease. First, we ran an initial cause of death model using CODEm, the cause of death ensemble model used throughout GBD, and an initial non-fatal model using DisMod-MR 2.1, the Bayesian meta-regression tool developed for GBD. The initial CODEm model used 14 990 site-years of data (ie, a unique combination of calendar year and country) as well as predictive covariates of SDI,^15^ cumulative cigarette consumption, health-care access and quality,^17^ education, and mean cholesterol level (a full list of predictive covariates is in the appendix). The initial DisMod-MR 2.1 model included settings of no remission (ie, no cure) and no incidence before the age of 20 years because the disease is exceptional before that age. We excluded all incidence data from the model, since we saw inconsistencies between the available prevalence and incidence data, and we considered measurement error less likely to occur with prevalence data than with incidence data. We let DisMod-MR 2.1 adjust medical claims data to correct for any systematic under-reporting and datapoints with case definitions that differed from the reference. Smoking prevalence and SDI were used as predictive covariates in the model.

We used these initial model results to identify countries with high-quality vital registration systems, age-standardised prevalence of more than five per 10 000, and a population of more than 1 million that also had the highest ratios of cause-specific mortality to prevalence, or highest likelihood to code to Parkinson's disease as a cause of death per prevalent case in the most recent year of estimates. For GBD 2016, these countries were Austria, Finland, and the USA. We then used the log-transformed ratios of cause-specific mortality rate to prevalence in 2016 to run a fixed-effects regression with dummy variables on age and sex. Because the ratio between cause-specific mortality rate and prevalence is equivalent to an excess mortality rate or excess rate of dying among people with Parkinson's disease compared with the general population, we used the results of this regression as input data for a second DisMod-MR 2.1 model. The excess mortality data obtained from the regression model were used as data for the entire 1990–2016 period and for every country except for the three used in the regression model, which retained their own data for 2016, and data for these countries were assumed to be constant over the entire time series. Apart from this addition of excess mortality data, the second DisMod-MR 2.1 model was identical to the initial model and used the same settings and covariates. We used the cause-specific mortality and prevalence results from this model as final outputs because they ensured consistency between the available non-fatal input data and the excess mortality rate in 2016 from the three countries most likely to code to Parkinson's disease as a cause of death.

### Severity and years lived with disability

To calculate years lived with disability (YLDs) for Parkinson's disease, we split the overall prevalence from the second DisMod-MR 2.1 model into three severity categories using data reporting on the Hoehn and Yahr stages.^18^, ^19^ We used 30 unique sources, covering nine of 21 world regions, and equated a score of 2·0 or less on the Hoehn and Yahr scale to mild Parkinson's disease, a score of 2·5–3·0 to moderate Parkinson's disease, and a score of 4·0–5·0 to severe Parkinson's disease (appendix). These data informed meta-analyses of the proportion of Parkinson's disease that is mild, moderate, and severe. We then used these proportions to split the overall prevalence of Parkinson's disease into the severity categories. Finally, we multiplied the prevalence of each severity category by severity-specific disability weights^20^ (see appendix for a detailed description) to calculate YLDs. YLDs were then corrected for comorbidity with a simulation that assigned all non-fatal outcomes to hypothetical individuals and adjusted disability in patients who had multiple conditions.

### Risk estimation

Of the 84 risks quantified in GBD 2016,^21^ only smoking was judged to have sufficient evidence for a relationship with Parkinson's disease, with smoking associated with decreased risk.^10^ The main sources of exposure data were population-based surveys. We used the estimates of exposure, relative risk, and a theoretical minimum level of exposure of zero lifetime cigarettes smoked to calculate population attributable fractions. Further information on risk factor calculations can be found in the GBD 2016 risk factors paper.^21^

### Compilation of results

We calculated years of life lost (YLLs) by multiplying the number of deaths in an age group by the remaining life expectancy in that age group, taken from the GBD standard life table.^22^ Disability-adjusted life-years (DALYs) were then calculated as the sum of YLLs and YLDs.

Through each computational step, uncertainty was propagated by sampling 1000 draws, which allowed us to combine the uncertainty from input data, corrections to the data, and residual non-sampling error. Uncertainty intervals (UIs) were defined as the 25th and 975th values of the ordered draws.

### Role of the funding source

The funder of the study had no role in study design, data collection, data analysis, data interpretation, or writing of the report. All authors had full access to the study data and had final responsibility for the decision to submit for publication.

## Results

The results of our analyses can be downloaded from the Global Health Data Exchange and Institute for Health Metrics and Evaluation (Seattle, WA, USA) results tools. Through the systematic analysis we identified 127 data sources on Parkinson's disease, including 91 sources on prevalence covering 16 of the 21 GBD world regions, 34 sources on incidence covering nine world regions, and 11 sources on mortality risk covering two world regions (appendix). The 11 sources on mortality risk are used in the non-fatal modelling process and therefore are marked as belonging to “non-fatal” in the Global Health Data Exchange input data tool. The 11 sources with data on mortality risk are easily identified by the words “mortality” or “survival” in the title. For prevalence, 40 (44·0%) studies were done in western Europe, nine (9·9%) in east Asia, seven (7·7%) each in high-income Asia Pacific, high-income North America, and North Africa and Middle East. 21 (23·0%) studies were from other regions, except for central Asia, central Latin America, tropical Latin America, central sub-Saharan Africa, and southern sub-Saharan Africa, for which no data were available.

**Figure 1:**
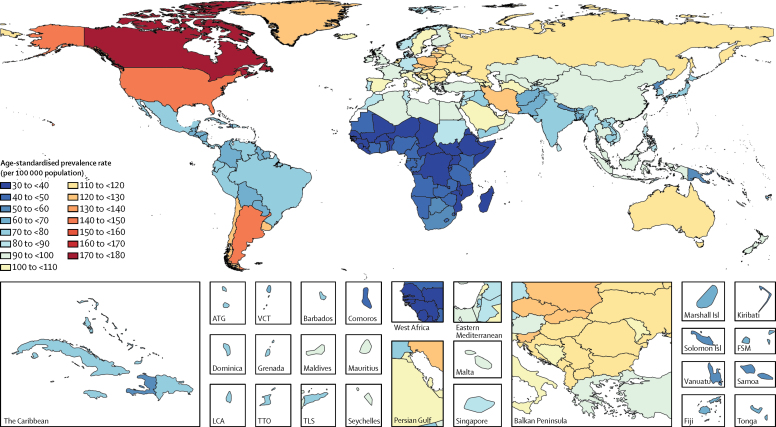
Age-standardised prevalence of Parkinson's disease per 100 000 population by location for both sexes, 2016 ATG=Antigua and Barbuda. FSM=Federated States of Micronesia. Isl=Islands. LCA=Saint Lucia. TLS=Timor-Leste. TTO=Trinidad and Tobago. VCT=Saint Vincent and the Grenadines.

In 2016, 6·1 million (95% UI 5·0–7·3) individuals worldwide had Parkinson's disease, of whom 2·9 million (47·5%) were women and 3·2 million (52·5%) were men. 2·1 million (34·4%) of these individuals were from high SDI countries, 3·1 million (50·8%) from high-middle or middle SDI countries, and 0·9 million (14·8%) from low-middle or low SDI countries (table). The number of individuals with Parkinson's disease in 2016 was 2·4 times higher than in 1990 (2·5 million, 95% UI 2·0–3·0). In 1990, 1·1 million (44·0%) cases were in high SDI countries, 1·1 million (44·0%) in high-middle or middle SDI countries, and 0·3 million (12·0%) in low-middle or low SDI countries. The increase in the number of patients with Parkinson's disease worldwide between 1990 and 2016 was not explained exclusively by an increasing number of older people, because global age-standardised prevalence rates increased by 21·7% (95% UI 18·1–25·3) from 1990 to 2016 compared with an increase of 74·3% (69·2–79·6) for crude prevalence rates. The increase in the number of patients with Parkinson's disease between 1990 and 2016 was less pronounced in high SDI countries (9·2%, 95% UI 5·5–13·2) than in other countries, and the largest increase was seen in middle SDI countries (59·8%, 53·2–66·1). The increase in age-standardised prevalence rates between 1990 and 2016 was similar in men (21·4%, 95% UI 17·6–24·9) and women (19·3%, 15·7–22·7). Age-standardised prevalence rates of Parkinson's disease by country varied greater than five times, with the highest rates generally in high-income North America and lowest rates in sub-Saharan Africa (figure 1).

**Table:** Deaths, prevalence, and DALYs for Parkinson's disease in 2016 and percentage change between 1990 and 2016 in age-standardised rates by location

	**Deaths**		**Prevalence**		**DALYs**	
	2016 counts	Percentage change in age-standardised rates, 1990–2016	2016 counts	Percentage change in age-standardised rates, 1990–2016	2016 counts	Percentage change in age-standardised rates, 1990–2016
**Global**	**211 296 (167 771 to 265 160)**	**19·5% (15·6 to 23·3)**	**6 062 893 (4 971 461 to 7 324 997)**	**21·7% (18·1 to 25·3)**	**3 234 514 (2 563 609 to 4 012 766)**	**22·1% (18·2 to 25·8)**
High SDI	84 911 (69 795 to 103 772)	11·3% (7·0 to 16·2)	2 052 069 (1 739 363 to 2 406 677)	9·2% (5·5 to 13·2)	1 128 768 (923 886 to 1 359 135)	9·9% (6·0 to 14·4)
High-middle SDI	44 111 (33 506 to 56 880)	13·8% (6·3 to 21·6)	1 328 576 (1 056 629 to 1 639 499)	20·3% (16·4 to 24·2)	682 750 (523 447 to 864 241)	17·1% (10·3 to 24·3)
Middle SDI	54 709 (42 446 to 69 607)	49·3% (42·7 to 55·8)	1 778 180 (1 434 399 to 2 166 529)	59·8% (53·2 to 66·1)	942 921 (738 016 to 1 174 509)	57·2% (50·4 to 63·8)
Low-middle SDI	23 409 (17 892 to 30 263)	45·4% (36·0 to 56·0)	786 869 (624 622 to 970 524)	31·6% (28·3 to 34·8)	409 620 (316 011 to 515 880)	42·6% (36·0 to 50·5)
Low SDI	4061 (3088 to 5273)	34·9% (27·0 to 43·4)	112 859 (88 680 to 141 337)	20·8% (18·3 to 23·5)	68 638 (52 894 to 87 401)	32·4% (26·4 to 39·5)
**High-income North America**	**30 461 (27 651 to 33 532)**	**25·7% (14·0 to 38·9)**	**811 354 (749 201 to 873 720)**	**12·8% (2·8 to 23·8)**	**414 699 (366 012 to 460 392)**	**18·7% (8·1 to 30·8)**
Canada	4343 (3131 to 5477)	45·4% (15·1 to 71·6)	103 903 (78 532 to 126 685)	43·0% (16·5 to 67·0)	58 911 (43 090 to 73 670)	44·5% (15·1 to 69·0)
Greenland	1 (1 to 2)	12·8% (−4·7 to 33·6)	45 (35 to 57)	12·7% (6·8 to 19·2)	24 (17 to 32)	13·0% (−3·5 to 32·3)
USA	26 117 (24 057 to 28 472)	22·4% (8·9 to 36·8)	707 158 (664 026 to 753 627)	9·5% (−0·8 to 21·8)	355 735 (320 852 to 392 813)	15·0% (3·4 to 28·7)
**Australasia**	**1979 (1510 to 2588)**	**16·1% (6·9 to 26·4)**	**47 265 (37 446 to 58 360)**	**9·0% (3·5 to 14·2)**	**27 109 (20 483 to 34 716)**	**10·9% (3·0 to 19·8)**
Australia	1721 (1314 to 2260)	15·5% (4·9 to 27·3)	41 016 (32 614 to 50 769)	8·2% (1·8 to 14·4)	23 497 (17 742 to 30 279)	10·1% (1·4 to 19·9)
New Zealand	258 (196 to 338)	18·5% (5·5 to 33·0)	6249 (4876 to 7728)	13·6% (7·5 to 19·8)	3612 (2736 to 4638)	14·9% (3·9 to 27·1)
**High-income Asia Pacific**	**13 181 (10 069 to 17 172)**	**11·9% (6·2 to 18·3)**	**316 347 (248 589 to 395 456)**	**21·2% (18·6 to 24·0)**	**174 232 (132 665 to 223 694)**	**16·5% (11·2 to 22·2)**
Brunei	5 (4 to 7)	17·9% (3·1 to 31·7)	180 (144 to 221)	12·5% (6·0 to 18·1)	95 (73 to 121)	17·0% (4·0 to 29·7)
Japan	10 936 (8270 to 14 260)	10·2% (5·7 to 14·8)	256 455 (201 529 to 321 563)	21·3% (18·6 to 24·2)	141 226 (107 717 to 181 551)	15·7% (11·7 to 19·5)
Singapore	165 (119 to 220)	11·3% (−8·3 to 35·9)	4166 (3324 to 5180)	16·2% (10·6 to 22·3)	2270 (1709 to 2923)	12·7% (−4·1 to 34·0)
South Korea	2075 (1459 to 2871)	24·6% (−3·9 to 59·9)	55 545 (43 464 to 68 533)	21·0% (14·9 to 28·1)	30 642 (22 126 to 41 397)	21·4% (−3·1 to 50·9)
**Western Europe**	**38 233 (29 203 to 50 366)**	**9·0% (0·5 to 17·3)**	**828 703 (652 541 to 1 036 222)**	**8·4% (0·5 to 15·1)**	**487 578 (369 862 to 629 443)**	**8·2% (0·0 to 15·7)**
Andorra	8 (6 to 10)	15·9% (−7·2 to 43·8)	154 (122 to 195)	12·1% (6·5 to 17·7)	94 (70 to 125)	14·1% (−7·2 to 38·0)
Austria	744 (543 to 1011)	15·1% (5·4 to 26·9)	15 891 (12 441 to 19 948)	14·2% (8·2 to 20·0)	9574 (6988 to 12 832)	14·6% (5·8 to 24·5)
Belgium	975 (732 to 1276)	15·5% (3·1 to 28·7)	20 862 (16 363 to 26 229)	12·4% (6·7 to 18·4)	12 338 (9301 to 15 866)	13·6% (3·2 to 25·3)
Cyprus	58 (43 to 75)	4·9% (−4·4 to 15·6)	1245 (982 to 1558)	16·3% (10·4 to 22·3)	757 (580 to 978)	6·0% (−3·0 to 15·4)
Denmark	411 (312 to 541)	56·7% (30·8 to 88·6)	9068 (7118 to 11 235)	45·9% (29·4 to 66·9)	5463 (4161 to 7061)	51·3% (28·8 to 79·4)
Finland	525 (384 to 714)	15·7% (2·6 to 28·8)	10 258 (8074 to 12 834)	5·8% (−3·6 to 13·7)	6935 (5102 to 9185)	10·3% (−0·8 to 22·0)
France	5798 (4370 to 7590)	−5·1% (−17·3 to 7·5)	120 455 (94 861 to 151 896)	−2·2% (−13·0 to 8·1)	70 410 (53 916 to 90 225)	−4·9% (−16·4 to 6·3)
Germany	7306 (5402 to 9675)	14·5% (1·9 to 28·9)	162 246 (126 379 to 203 964)	11·5% (2·8 to 19·7)	96 664 (73 054 to 127 109)	12·6% (0·7 to 25·0)
Greece	1066 (801 to 1387)	11·6% (1·2 to 22·6)	22 837 (17 855 to 28 778)	13·2% (7·8 to 18·9)	13 376 (10 072 to 17 216)	11·5% (3·0 to 21·3)
Iceland	22 (17 to 29)	20·4% (8·2 to 33·4)	474 (375 to 591)	13·4% (8·2 to 18·5)	287 (217 to 373)	17·4% (6·7 to 28·3)
Ireland	251 (187 to 335)	18·0% (3·8 to 34·1)	6001 (4712 to 7491)	17·2% (9·9 to 24·0)	3451 (2607 to 4524)	16·4% (3·8 to 30·5)
Israel	411 (304 to 544)	−4·8% (−22·5 to 13·6)	9395 (7477 to 11 858)	−4·1% (−10·7 to 2·4)	5338 (4016 to 6964)	−5·6% (−21·1 to 10·5)
Italy	6520 (4878 to 8605)	−5·5% (−20·8 to 12·0)	144 606 (113 316 to 180 277)	−3·4% (−16·1 to 9·5)	82 834 (62 455 to 108 059)	−4·6% (−19·5 to 12·2)
Luxembourg	40 (30 to 53)	18·1% (5·9 to 31·4)	873 (681 to 1091)	13·4% (7·9 to 19·9)	520 (395 to 673)	16·0% (5·5 to 27·4)
Malta	29 (21 to 39)	15·3% (−2·2 to 36·3)	720 (561 to 906)	15·4% (10·4 to 22·8)	418 (311 to 550)	15·4% (−0·2 to 33·6)
Netherlands	1467 (1099 to 1920)	−6·0% (−22·7 to 11·7)	33 297 (25 931 to 41 654)	−7·5% (−22·1 to 6·1)	19 621 (14 640 to 25 313)	−6·7% (−23·9 to 9·2)
Norway	342 (258 to 447)	93·0% (54·2 to 141·0)	7517 (5900 to 9463)	87·1% (54·9 to 122·6)	4412 (3338 to 5691)	93·9% (54·9 to 137·2)
Portugal	842 (634 to 1119)	34·3% (13·1 to 59·4)	18 496 (14 530 to 23 206)	31·9% (11·5 to 54·4)	10 902 (8249 to 14 165)	32·9% (11·2 to 56·9)
Spain	4363 (3322 to 5775)	0·6% (−12·6 to 15·7)	92 971 (73 044 to 116 691)	8·0% (−4·3 to 20·5)	54 175 (41 280 to 70 222)	4·9% (−8·2 to 19·3)
Sweden	921 (679 to 1202)	18·3% (6·1 to 33·8)	19 776 (15 538 to 24 631)	13·6% (9·0 to 18·9)	11 805 (8933 to 15 111)	15·1% (4·4 to 27·7)
Switzerland	695 (499 to 942)	13·9% (−8·9 to 42·4)	14 979 (11 761 to 18 750)	10·3% (4·4 to 16·0)	8857 (6553 to 11 759)	10·8% (−7·9 to 34·4)
UK	5438 (4194 to 7099)	32·5% (28·5 to 36·9)	115 846 (91 722 to 144 139)	22·3% (20·0 to 24·7)	69 262 (53 335 to 88 203)	26·3% (23·0 to 30·1)
**Southern Latin America**	**4149 (3109 to 5407)**	**3·1% (−9·1 to 14·7)**	**100 190 (77 965 to 125 392)**	**5·4% (−4·1 to 14·2)**	**57 932 (43 522 to 73 989)**	**4·3% (−6·6 to 14·9)**
Argentina	2798 (2099 to 3623)	0·1% (−13·4 to 13·8)	68 048 (52 574 to 85 157)	2·5% (−8·5 to 13·5)	39 297 (29 708 to 49 971)	1·8% (−10·2 to 14·6)
Chile	1064 (756 to 1437)	16·5% (−7·1 to 43·0)	25 845 (20 232 to 32 298)	19·9% (12·8 to 27·5)	14 860 (10 643 to 19 579)	17·1% (−2·7 to 40·4)
Uruguay	287 (216 to 375)	7·1% (−2·9 to 18·9)	6289 (4860 to 7952)	10·9% (2·8 to 18·7)	3775 (2870 to 4861)	8·2% (−1·2 to 19·2)
**Eastern Europe**	**12 866 (9222 to 17 122)**	**8·5% (−9·1 to 29·9)**	**365 078 (282 400 to 459 433)**	**6·9% (2·4 to 11·4)**	**197 660 (141 621 to 259 341)**	**8·6% (−6·4 to 26·8)**
Belarus	599 (431 to 820)	10·8% (−6·2 to 29·9)	16 588 (12 933 to 20 675)	9·0% (3·6 to 14·1)	9005 (6559 to 11 905)	10·8% (−3·2 to 26·8)
Estonia	121 (88 to 158)	5·6% (−10·3 to 22·0)	3078 (2372 to 3877)	1·5% (−12·1 to 12·6)	1722 (1259 to 2228)	3·2% (−12·0 to 18·6)
Latvia	178 (127 to 237)	7·7% (−4·6 to 21·1)	4613 (3553 to 5819)	7·6% (2·3 to 14·2)	2586 (1905 to 3373)	8·0% (−3·1 to 20·0)
Lithuania	266 (196 to 349)	9·2% (−0·4 to 19·6)	6775 (5234 to 8545)	8·8% (3·1 to 14·9)	3795 (2819 to 4927)	9·6% (0·9 to 18·7)
Moldova	161 (120 to 212)	5·9% (−6·0 to 20·5)	4825 (3785 to 6006)	6·5% (1·6 to 12·3)	2575 (1932 to 3332)	6·9% (−3·4 to 19·3)
Russia	8516 (5836 to 12 064)	9·0% (−16·5 to 41·7)	244 559 (189 528 to 307 585)	7·1% (0·9 to 13·6)	131 691 (91 670 to 180 532)	9·0% (−12·2 to 36·2)
Ukraine	3025 (2094 to 4191)	6·9% (−12·3 to 32·3)	84 640 (65 434 to 106 094)	5·7% (0·6 to 10·7)	46 286 (32 447 to 62 092)	7·1% (−9·3 to 27·4)
**Central Europe**	**9061 (6794 to 11 883)**	**8·6% (3·9 to 13·4)**	**231 329 (179 712 to 291 637)**	**10·2% (7·6 to 12·8)**	**131 027 (99 413 to 168 732)**	**9·4% (5·1 to 13·7)**
Albania	150 (111 to 202)	10·9% (−4·3 to 27·1)	4067 (3131 to 5154)	14·0% (7·6 to 20·5)	2295 (1709 to 3026)	12·4% (−0·7 to 26·3)
Bosnia and Herzegovina	253 (186 to 334)	16·0% (−0·9 to 36·2)	6631 (5127 to 8317)	19·5% (13·7 to 25·5)	3739 (2788 to 4814)	18·0% (2·9 to 35·5)
Bulgaria	665 (485 to 898)	−5·3% (−21·7 to 11·5)	16 915 (13 099 to 21 383)	0·8% (−10·7 to 11·1)	9672 (7100 to 12 864)	−2·3% (−17·9 to 12·8)
Croatia	402 (300 to 533)	7·9% (−6·3 to 21·6)	9662 (7485 to 12 183)	7·9% (2·5 to 13·7)	5653 (4220 to 7339)	8·6% (−3·8 to 20·9)
Czech Republic	866 (647 to 1133)	7·6% (−1·5 to 17·6)	22 651 (17 359 to 28 690)	9·3% (4·0 to 15·4)	12 719 (9558 to 16 483)	7·5% (−0·9 to 16·5)
Hungary	822 (608 to 1074)	8·3% (−3·9 to 21·5)	20 908 (16 195 to 26 302)	9·5% (4·3 to 16·1)	11 898 (8915 to 15 343)	9·0% (−1·5 to 21·0)
Macedonia	108 (80 to 140)	5·9% (−3·1 to 15·7)	3021 (2340 to 3787)	7·7% (2·3 to 13·4)	1705 (1280 to 2213)	6·8% (−2·0 to 15·8)
Montenegro	39 (29 to 51)	10·9% (−2·5 to 25·1)	1035 (799 to 1307)	8·4% (2·7 to 14·3)	582 (440 to 752)	10·3% (−1·2 to 22·4)
Poland	2943 (2195 to 3902)	13·7% (2·5 to 25·5)	74 905 (58 130 to 93 685)	14·2% (8·6 to 20·4)	41 955 (31 278 to 54 100)	14·0% (4·0 to 23·8)
Romania	1605 (1201 to 2110)	8·1% (−2·4 to 20·3)	40 517 (31 427 to 50 995)	10·2% (4·5 to 15·5)	23 144 (17 467 to 30 057)	9·2% (0·2 to 20·0)
Serbia	650 (482 to 832)	8·7% (−1·5 to 20·8)	16 702 (12 943 to 20 877)	7·6% (2·5 to 12·6)	9540 (7187 to 12 219)	8·9% (−0·1 to 19·5)
Slovakia	358 (264 to 466)	8·0% (−4·4 to 21·2)	9523 (7411 to 11 952)	9·7% (4·7 to 14·9)	5368 (3965 to 6965)	8·6% (−2·2 to 20·0)
Slovenia	200 (150 to 264)	6·7% (−7·5 to 20·9)	4792 (3697 to 6028)	9·7% (4·0 to 15·6)	2755 (2061 to 3601)	7·4% (−5·0 to 20·5)
**Central Asia**	**1833 (1353 to 2401)**	**10·5% (4·6 to 16·9)**	**56 062 (44 137 to 69 550)**	**10·4% (7·9 to 12·9)**	**29 509 (22 429 to 37 368)**	**10·7% (5·4 to 16·4)**
Armenia	142 (106 to 188)	13·5% (1·8 to 27·3)	3727 (2918 to 4629)	10·7% (4·1 to 17·1)	2058 (1565 to 2663)	13·1% (2·8 to 24·7)
Azerbaijan	235 (165 to 318)	15·1% (−1·6 to 35·6)	7307 (5748 to 9051)	11·9% (7·0 to 17·2)	3836 (2790 to 4989)	13·8% (−1·0 to 31·1)
Georgia	236 (171 to 315)	8·6% (−7·2 to 24·9)	5900 (4565 to 7397)	5·2% (0·5 to 11·3)	3370 (2496 to 4368)	8·3% (−5·7 to 23·2)
Kazakhstan	397 (288 to 530)	5·5% (−9·4 to 24·9)	13 372 (10 530 to 16 557)	9·0% (3·4 to 14·5)	6764 (4973 to 8711)	6·3% (−6·9 to 22·6)
Kyrgyzstan	90 (67 to 117)	6·7% (−2·8 to 17·7)	2802 (2209 to 3474)	5·2% (0·3 to 10·6)	1465 (1107 to 1854)	6·9% (−1·5 to 17·1)
Mongolia	40 (29 to 53)	5·2% (−9·0 to 21·2)	1353 (1067 to 1694)	11·2% (5·4 to 17·7)	704 (523 to 916)	5·9% (−6·7 to 19·6)
Tajikistan	92 (69 to 121)	12·0% (−1·9 to 30·7)	2884 (2269 to 3573)	8·9% (3·5 to 14·9)	1533 (1164 to 1961)	12·0% (−0·8 to 28·4)
Turkmenistan	79 (60 to 103)	14·2% (4·5 to 24·9)	2711 (2144 to 3363)	18·3% (12·3 to 23·9)	1370 (1044 to 1751)	16·2% (7·3 to 25·4)
Uzbekistan	522 (387 to 681)	14·0% (2·4 to 25·7)	16 006 (12 659 to 19 798)	15·3% (9·6 to 21·8)	8409 (6379 to 10 644)	15·5% (4·7 to 26·4)
**Central Latin America**	**4246 (3249 to 5442)**	**13·2% (9·2 to 17·2)**	**129 124 (102 593 to 159 008)**	**16·6% (14·6 to 18·5)**	**67 023 (51 781 to 84 193)**	**14·8% (11·1 to 18·5)**
Colombia	796 (607 to 1022)	12·2% (1·4 to 25·2)	25 930 (20 527 to 32 111)	15·5% (9·8 to 20·8)	13 140 (10 059 to 16 860)	13·4% (3·5 to 25·1)
Costa Rica	110 (83 to 143)	12·8% (2·4 to 24·4)	3230 (2532 to 4003)	15·3% (10·0 to 21·2)	1700 (1300 to 2192)	14·0% (4·6 to 24·3)
El Salvador	128 (97 to 168)	13·8% (0·9 to 28·3)	3436 (2680 to 4268)	18·6% (12·2 to 25·1)	1902 (1440 to 2462)	13·7% (2·9 to 26·5)
Guatemala	182 (130 to 244)	15·7% (−8·2 to 41·6)	5194 (4068 to 6433)	18·0% (12·3 to 23·8)	2825 (2078 to 3721)	15·7% (−5·8 to 37·6)
Honduras	107 (76 to 146)	19·8% (−3·2 to 47·4)	2741 (2166 to 3383)	18·4% (12·8 to 24·4)	1626 (1168 to 2140)	18·7% (−2·0 to 43·7)
Mexico	2299 (1752 to 2952)	14·4% (10·3 to 18·6)	68 715 (54 711 to 83 874)	17·7% (15·9 to 19·6)	35 633 (27 612 to 44 856)	16·4% (12·8 to 20·0)
Nicaragua	77 (58 to 102)	13·8% (−1·7 to 32·1)	2273 (1799 to 2788)	15·9% (10·6 to 22·0)	1185 (908 to 1519)	14·8% (1·6 to 30·4)
Panama	83 (62 to 108)	11·4% (−3·3 to 29·4)	2369 (1878 to 2906)	14·3% (8·5 to 19·4)	1255 (949 to 1629)	12·2% (−0·6 to 26·9)
Venezuela	464 (335 to 613)	9·3% (−6·2 to 28·6)	15 235 (12 126 to 18 584)	14·0% (6·9 to 19·9)	7758 (5734 to 10 079)	11·0% (−2·9 to 29·0)
**Andean Latin America**	**1093 (832 to 1450)**	**15·3% (3·7 to 28·5)**	**30 717 (24 372 to 37 972)**	**13·0% (9·1 to 16·4)**	**16 698 (12 634 to 21 390)**	**14·8% (4·7 to 26·3)**
Bolivia	202 (147 to 269)	21·1% (3·1 to 43·7)	5114 (4011 to 6330)	14·1% (8·8 to 19·8)	3003 (2237 to 3871)	19·2% (3·0 to 38·5)
Ecuador	295 (224 to 383)	10·8% (0·7 to 22·5)	8688 (6872 to 10 735)	13·6% (8·5 to 19·3)	4589 (3519 to 5833)	11·2% (2·3 to 21·8)
Peru	596 (436 to 806)	15·9% (−4·6 to 37·7)	16 915 (13 398 to 20 887)	12·3% (6·8 to 18·0)	9106 (6795 to 11 952)	15·2% (−2·4 to 34·8)
**Caribbean**	**1169 (883 to 1515)**	**10·6% (3·6 to 17·5)**	**31 751 (25 123 to 39 315)**	**11·4% (7·6 to 14·9)**	**17 253 (13 197 to 22 090)**	**11·3% (5·5 to 17·4)**
Antigua and Barbuda	2 (1 to 3)	14·5% (0·9 to 30·7)	57 (45 to 70)	12·3% (6·8 to 18·0)	31 (23 to 39)	13·3% (1·7 to 27·1)
The Bahamas	9 (7 to 12)	11·5% (−1·8 to 25·6)	262 (206 to 325)	9·4% (3·4 to 14·1)	145 (109 to 184)	10·5% (−1·1 to 22·2)
Barbados	12 (9 to 17)	18·6% (5·6 to 32·2)	314 (247 to 392)	11·3% (5·5 to 16·7)	180 (136 to 235)	16·2% (5·3 to 27·7)
Belize	4 (3 to 5)	30·4% (12·1 to 48·3)	105 (82 to 130)	20·1% (13·8 to 25·9)	60 (45 to 76)	28·9% (13·0 to 46·0)
Bermuda	2 (1 to 3)	12·8% (−3·1 to 30·5)	52 (41 to 64)	10·7% (6·0 to 15·9)	30 (22 to 38)	11·9% (−2·2 to 26·1)
Cuba	507 (381 to 662)	7·9% (−4·4 to 20·2)	12 678 (9985 to 15 908)	9·0% (2·0 to 16·5)	7203 (5459 to 9294)	9·0% (−2·0 to 19·9)
Dominica	2 (1 to 3)	17·2% (3·5 to 33·8)	51 (40 to 63)	15·5% (10·1 to 21·0)	29 (22 to 37)	16·5% (4·1 to 31·3)
Dominican Republic	202 (149 to 264)	13·8% (−3·2 to 30·9)	5456 (4317 to 6690)	17·5% (11·5 to 23·6)	2999 (2282 to 3831)	14·6% (−0·3 to 30·6)
Grenada	2 (2 to 3)	29·3% (12·9 to 45·8)	58 (46 to 72)	22·7% (17·1 to 28·6)	34 (26 to 43)	28·3% (14·1 to 43·2)
Guyana	8 (6 to 10)	16·8% (3·8 to 30·2)	297 (235 to 367)	15·1% (10·0 to 20·3)	152 (116 to 193)	16·0% (4·7 to 28·1)
Haiti	103 (74 to 139)	21·7% (4·3 to 41·3)	3025 (2368 to 3805)	14·5% (8·4 to 20·2)	1738 (1258 to 2333)	20·7% (4·3 to 39·2)
Jamaica	81 (60 to 107)	20·8% (3·1 to 42·6)	1949 (1544 to 2425)	13·7% (7·8 to 19·2)	1123 (827 to 1467)	19·6% (3·4 to 38·1)
Puerto Rico	181 (136 to 235)	14·1% (2·4 to 26·9)	4300 (3392 to 5366)	12·2% (7·1 to 18·6)	2478 (1872 to 3177)	13·5% (3·0 to 24·7)
Saint Lucia	5 (4 to 7)	21·3% (9·6 to 33·1)	128 (101 to 160)	18·0% (12·8 to 25·4)	73 (56 to 93)	20·8% (10·7 to 31·8)
Saint Vincent and the Grenadines	2 (2 to 3)	17·1% (4·5 to 31·4)	62 (49 to 77)	18·6% (12·6 to 24·6)	34 (26 to 44)	17·9% (6·7 to 30·3)
Suriname	10 (8 to 13)	20·1% (8·4 to 33·9)	285 (225 to 351)	14·0% (8·5 to 20·1)	161 (123 to 206)	17·8% (6·9 to 29·5)
Trinidad and Tobago	32 (24 to 41)	10·8% (1·2 to 20·9)	987 (779 to 1221)	13·2% (7·2 to 18·8)	522 (398 to 663)	11·3% (2·3 to 20·6)
Virgin Islands	5 (4 to 7)	10·3% (−1·4 to 24·1)	143 (112 to 181)	12·8% (7·5 to 18·7)	83 (62 to 108)	10·3% (−0·5 to 22·9)
**Tropical Latin America**	**4132 (3149 to 5331)**	**15·2% (11·3 to 19·9)**	**131 748 (104 807 to 162 882)**	**16·5% (14·3 to 18·8)**	**67 778 (52 122 to 85 976)**	**15·2% (11·8 to 19·2)**
Brazil	4033 (3074 to 5199)	15·0% (11·1 to 19·6)	128 836 (102 469 to 159 395)	16·4% (14·2 to 18·7)	66 204 (50 914 to 84 027)	15·0% (11·6 to 19·1)
Paraguay	98 (74 to 129)	23·4% (8·0 to 39·4)	2912 (2300 to 3602)	19·4% (13·7 to 25·2)	1573 (1189 to 2015)	23·1% (10·7 to 37·0)
**East Asia**	**41 584 (32 292 to 53 090)**	**77·4% (64·4 to 89·0)**	**1 451 650 (1 162 770 to 1 793 630)**	**109·4% (94·2 to 123·8)**	**734 156 (567 971 to 920 610)**	**93·8% (80·1 to 107·0)**
China	40 012 (31 132 to 51 074)	82·7% (68·3 to 95·3)	1 407 701 (1 126 570 to 1 738 833)	115·7% (99·5 to 131·0)	710 041 (549 539 to 890 276)	100·4% (85·3 to 114·9)
North Korea	418 (310 to 545)	15·1% (0·1 to 35·4)	14 098 (11 067 to 17 482)	6·0% (1·2 to 10·4)	7527 (5649 to 9693)	14·4% (1·5 to 30·5)
Taiwan (province of China)	1154 (868 to 1540)	−20·1% (−32·7 to −6·6)	29 851 (23 788 to 36 740)	2·0% (−7·8 to 9·2)	16 588 (12 664 to 21 336)	−17·2% (−29·5 to −5·5)
**Southeast Asia**	**11 900 (9275 to 14 966)**	**34·2% (26·6 to 43·6)**	**409 655 (338 902 to 493 631)**	**24·7% (21·1 to 29·1)**	**213 332 (169 151 to 262 446)**	**31·4% (25·2 to 38·5)**
Cambodia	172 (130 to 217)	57·6% (38·1 to 88·1)	5768 (4623 to 7126)	26·6% (20·8 to 32·6)	3302 (2563 to 4119)	46·9% (30·9 to 68·6)
Indonesia	3490 (2682 to 4487)	55·1% (39·6 to 76·0)	146 236 (117 531 to 178 755)	21·7% (19·1 to 24·2)	70 145 (54 835 to 88 069)	43·4% (32·7 to 56·7)
Laos	62 (47 to 81)	48·0% (32·0 to 71·5)	2305 (1851 to 2826)	26·0% (19·9 to 32·3)	1190 (911 to 1512)	42·8% (29·5 to 60·6)
Malaysia	514 (386 to 672)	19·9% (8·5 to 32·7)	19 586 (15 697 to 23 908)	26·4% (20·9 to 32·7)	9694 (7546 to 12 275)	19·1% (9·4 to 29·0)
Maldives	6 (4 to 8)	25·8% (2·7 to 53·4)	187 (152 to 227)	25·1% (19·7 to 31·1)	96 (71 to 123)	25·8% (6·3 to 51·0)
Mauritius	34 (26 to 45)	19·8% (5·9 to 35·2)	1224 (971 to 1492)	21·5% (16·0 to 27·0)	606 (465 to 765)	18·1% (6·2 to 31·8)
Myanmar	1129 (859 to 1471)	45·7% (29·6 to 64·5)	28 152 (22 550 to 34 703)	29·4% (23·4 to 35·9)	19 732 (15 161 to 25 096)	41·2% (26·7 to 57·4)
Philippines	1132 (851 to 1462)	17·0% (3·1 to 31·4)	45 978 (37 005 to 56 836)	16·9% (11·3 to 22·8)	22 431 (17 069 to 28 411)	17·6% (5·5 to 30·5)
Sri Lanka	558 (402 to 758)	8·2% (−11·3 to 30·0)	17 814 (14 263 to 21 949)	20·5% (15·0 to 26·8)	9475 (6993 to 12 384)	11·9% (−5·4 to 31·9)
Seychelles	2 (2 to 3)	16·0% (3·2 to 29·1)	76 (62 to 92)	19·0% (13·5 to 25·1)	39 (30 to 49)	15·1% (4·0 to 26·6)
Thailand	2400 (1917 to 2980)	25·2% (8·7 to 46·6)	76 568 (66 494 to 86 991)	31·7% (16·3 to 52·6)	40 432 (32 376 to 48 959)	27·6% (12·3 to 46·0)
Timor-Leste	13 (9 to 18)	50·6% (21·4 to 93·9)	454 (357 to 565)	26·6% (21·1 to 33·4)	250 (180 to 334)	44·8% (19·1 to 77·9)
Vietnam	2389 (1819 to 3083)	29·9% (11·8 to 51·0)	64 452 (52 241 to 78 194)	25·2% (19·6 to 30·9)	35 840 (28 180 to 44 837)	27·0% (10·9 to 44·2)
**Oceania**	**62 (48 to 80)**	**6·5% (−1·8 to 16·2)**	**2687 (2124 to 3321)**	**14·8% (11·7 to 18·2)**	**1243 (963 to 1560)**	**9·1% (1·1 to 18·8)**
American Samoa	1 (0 to 1)	−0·8% (−13·9 to 15·6)	21 (17 to 26)	11·5% (6·4 to 16·8)	10 (8 to 13)	1·3% (−11·4 to 15·4)
Federated States of Micronesia	1 (1 to 2)	13·4% (−3·6 to 36·1)	32 (25 to 39)	19·1% (12·6 to 25·1)	20 (15 to 26)	14·5% (−2·5 to 37·3)
Fiji	10 (7 to 14)	5·4% (−14·9 to 28·7)	386 (306 to 478)	14·7% (9·6 to 20·1)	195 (145 to 256)	7·5% (−11·3 to 30·6)
Guam	4 (3 to 5)	5·3% (−7·1 to 18·3)	116 (91 to 144)	9·8% (5·2 to 15·8)	64 (48 to 82)	5·8% (−5·6 to 17·4)
Kiribati	1 (1 to 1)	28·3% (11·2 to 47·1)	26 (20 to 32)	15·9% (10·5 to 22·2)	18 (13 to 23)	25·6% (9·7 to 41·2)
Marshall Islands	0 (0 to 1)	−4·7% (−16·4 to 8·8)	17 (14 to 22)	13·3% (6·7 to 20·2)	8 (6 to 10)	−1·9% (−12·6 to 10·5)
Northern Mariana Islands	0 (0 to 1)	8·1% (−9·6 to 30·3)	21 (16 to 26)	14·0% (8·9 to 20·2)	9 (7 to 12)	9·5% (−6·6 to 29·2)
Papua New Guinea	34 (25 to 44)	18·9% (4·1 to 37·3)	1414 (1114 to 1760)	15·7% (10·8 to 20·9)	692 (526 to 885)	16·9% (3·3 to 34·0)
Samoa	3 (2 to 4)	8·5% (−6·4 to 26·0)	67 (53 to 84)	13·2% (7·8 to 19·4)	44 (33 to 56)	9·1% (−4·9 to 25·1)
Solomon Islands	4 (3 to 5)	12·7% (−1·1 to 29·3)	120 (94 to 148)	12·9% (6·9 to 19·4)	74 (55 to 95)	13·2% (−0·2 to 28·8)
Tonga	2 (1 to 2)	9·8% (−6·4 to 28·9)	41 (32 to 51)	13·9% (7·8 to 20·5)	25 (19 to 32)	11·0% (−3·8 to 29·3)
Vanuatu	2 (2 to 3)	14·6% (−0·4 to 31·0)	69 (54 to 85)	14·4% (8·8 to 20·9)	42 (31 to 54)	14·8% (0·6 to 30·5)
**North Africa and Middle East**	**9460 (7348 to 12 233)**	**41·9% (33·2 to 51·7)**	**297 861 (239 654 to 367 829)**	**41·4% (37·3 to 46·0)**	**153 897 (120 636 to 193 371)**	**42·8% (35·2 to 51·0)**
Afghanistan	183 (139 to 235)	37·5% (22·1 to 57·9)	5813 (4574 to 7275)	27·0% (20·2 to 35·4)	3436 (2641 to 4334)	36·2% (22·9 to 52·7)
Algeria	943 (714 to 1243)	42·4% (27·1 to 59·6)	24 250 (19 187 to 29 987)	38·0% (31·1 to 46·1)	14 128 (10 823 to 18 044)	42·4% (28·8 to 57·3)
Bahrain	11 (8 to 15)	26·4% (3·5 to 54·3)	480 (383 to 603)	29·0% (23·0 to 35·2)	226 (170 to 296)	25·9% (5·4 to 49·7)
Egypt	1436 (1075 to 1860)	40·2% (22·7 to 61·2)	48 694 (39 464 to 59 862)	40·7% (33·0 to 49·8)	24 460 (18 678 to 31 133)	41·0% (25·5 to 59·9)
Iran	1811 (1343 to 2381)	61·2% (35·4 to 96·4)	59 590 (48 749 to 73 996)	58·4% (49·1 to 71·0)	30 138 (23 315 to 38 332)	62·5% (39·2 to 92·4)
Iraq	299 (224 to 391)	23·3% (1·4 to 43·6)	9777 (7735 to 12 063)	22·7% (16·2 to 29·7)	5204 (3940 to 6679)	23·5% (3·1 to 42·8)
Jordan	83 (59 to 112)	27·1% (1·4 to 58·2)	2589 (2084 to 3172)	28·7% (18·8 to 37·9)	1353 (999 to 1763)	26·0% (2·9 to 53·6)
Kuwait	27 (19 to 37)	42·8% (9·0 to 86·7)	1280 (1019 to 1622)	35·7% (28·4 to 44·1)	572 (417 to 770)	40·5% (10·8 to 78·1)
Lebanon	168 (125 to 224)	19·7% (−1·0 to 46·3)	5114 (4051 to 6360)	33·9% (26·5 to 41·8)	2524 (1920 to 3280)	20·8% (2·1 to 44·6)
Libya	105 (80 to 136)	38·0% (19·4 to 58·7)	3109 (2499 to 3866)	42·2% (31·4 to 54·7)	1718 (1322 to 2203)	40·6% (23·3 to 59·6)
Morocco	695 (528 to 908)	57·3% (37·5 to 102·4)	20 893 (16 569 to 26 080)	39·7% (32·4 to 46·9)	10 968 (8401 to 13 728)	54·2% (38·0 to 84·4)
Oman	39 (30 to 50)	63·4% (43·1 to 87·5)	1496 (1200 to 1859)	74·3% (65·0 to 84·5)	724 (567 to 910)	67·9% (49·3 to 89·6)
Palestine	34 (25 to 43)	18·2% (2·9 to 35·0)	1143 (907 to 1419)	19·8% (13·7 to 26·0)	595 (461 to 745)	19·7% (5·9 to 34·6)
Qatar	11 (7 to 16)	32·5% (−1·3 to 76·5)	562 (441 to 714)	34·0% (26·8 to 41·6)	255 (179 to 354)	33·7% (3·2 to 75·8)
Saudi Arabia	337 (263 to 432)	65·7% (43·7 to 94·7)	12 853 (10 251 to 15 936)	65·1% (58·8 to 71·4)	6126 (4814 to 7779)	67·5% (48·4 to 92·1)
Sudan	349 (261 to 455)	53·2% (38·6 to 72·5)	11 758 (9351 to 14 608)	38·2% (31·8 to 46·6)	6097 (4668 to 7754)	50·4% (37·8 to 65·3)
Syria	233 (178 to 303)	41·8% (27·4 to 56·7)	7409 (5926 to 9203)	40·9% (33·7 to 47·8)	3803 (2945 to 4844)	42·6% (29·4 to 55·3)
Tunisia	321 (237 to 430)	40·0% (16·2 to 65·5)	8450 (6790 to 10 486)	44·9% (36·3 to 54·3)	4697 (3568 to 6052)	43·0% (21·4 to 65·8)
Turkey	2160 (1603 to 2865)	25·6% (6·8 to 48·0)	63 708 (50 912 to 79 341)	33·8% (27·0 to 40·9)	32 482 (24 631 to 42 095)	27·8% (11·0 to 46·7)
United Arab Emirates	39 (29 to 51)	48·6% (18·7 to 86·5)	2498 (1953 to 3181)	41·4% (34·5 to 49·4)	1101 (809 to 1473)	50·5% (21·1 to 84·5)
Yemen	178 (134 to 230)	60·9% (41·2 to 86·1)	6160 (4866 to 7739)	45·6% (38·0 to 54·0)	3262 (2521 to 4155)	58·1% (40·0 to 79·7)
**South Asia**	**21 007 (15 942 to 27 123)**	**48·3% (36·8 to 62·4)**	**696 108 (552 987 to 860 047)**	**29·2% (25·6 to 32·8)**	**364 282 (281 631 to 460 552)**	**43·8% (35·9 to 53·4)**
Bangladesh	1501 (1138 to 1953)	−10·2% (−22·6 to 6·2)	54 198 (42 488 to 67 532)	25·0% (18·8 to 31·7)	25 363 (19 435 to 32 110)	−3·8% (−15·8 to 9·8)
Bhutan	11 (8 to 15)	43·5% (21·0 to 73·5)	288 (227 to 357)	35·8% (28·9 to 43·7)	171 (129 to 222)	41·1% (21·1 to 66·1)
India	17 539 (13 317 to 22 637)	55·8% (42·3 to 71·8)	575 946 (458 316 to 712 213)	29·7% (25·9 to 33·5)	305 274 (235 390 to 385 725)	49·6% (40·9 to 60·1)
Nepal	319 (236 to 419)	66·7% (38·1 to 107·3)	9445 (7390 to 11 806)	29·5% (23·2 to 36·4)	5449 (4071 to 7009)	54·5% (33·8 to 82·1)
Pakistan	1637 (1213 to 2148)	43·8% (23·6 to 70·0)	56 231 (43 998 to 70 068)	27·9% (21·7 to 35·7)	28 025 (21 124 to 36 119)	39·5% (22·1 to 59·7)
**Southern sub-Saharan Africa**	**756 (577 to 988)**	**25·6% (16·4 to 36·8)**	**20 980 (16 480 to 26 027)**	**14·3% (12·0 to 16·6)**	**11 750 (9090 to 14 869)**	**23·5% (16·2 to 33·0)**
Botswana	15 (8 to 23)	26·3% (−23·0 to 69·2)	460 (361 to 569)	22·0% (15·3 to 28·8)	264 (159 to 384)	25·3% (−18·4 to 63·7)
Lesotho	14 (10 to 19)	17·2% (−5·3 to 44·2)	397 (312 to 501)	18·0% (11·6 to 24·5)	223 (161 to 295)	16·8% (−4·2 to 41·0)
Namibia	16 (10 to 23)	25·7% (−8·4 to 55·2)	442 (349 to 551)	20·2% (13·4 to 28·0)	272 (178 to 374)	23·6% (−6·4 to 50·5)
South Africa	594 (454 to 787)	21·3% (12·5 to 31·6)	17 305 (13 650 to 21 444)	14·2% (11·7 to 16·7)	9245 (7128 to 11 715)	19·9% (12·7 to 28·2)
Swaziland	7 (4 to 10)	11·2% (−15·0 to 40·7)	215 (168 to 270)	15·4% (8·7 to 22·4)	118 (77 to 164)	11·5% (−11·3 to 38·8)
Zimbabwe	110 (78 to 146)	55·9% (25·6 to 143·8)	2162 (1701 to 2685)	8·5% (2·7 to 14·3)	1628 (1189 to 2120)	47·0% (21·1 to 117·4)
**Western sub-Saharan Africa**	**1578 (1170 to 2059)**	**22·6% (13·5 to 32·1)**	**44 230 (34 637 to 55 905)**	**15·9% (12·6 to 19·3)**	**27 359 (20 483 to 35 211)**	**21·7% (13·2 to 30·5)**
Benin	47 (35 to 62)	15·6% (2·3 to 31·1)	1196 (932 to 1541)	14·1% (7·5 to 20·2)	811 (610 to 1058)	16·2% (4·0 to 29·4)
Burkina Faso	59 (44 to 79)	11·6% (−0·9 to 29·1)	1537 (1199 to 1939)	14·4% (8·1 to 21·8)	1023 (777 to 1308)	11·3% (0·1 to 26·4)
Cameroon	147 (105 to 195)	15·5% (−1·4 to 34·6)	3077 (2420 to 3870)	11·6% (6·1 to 16·9)	2268 (1646 to 2958)	15·3% (−0·7 to 33·6)
Cape Verde	6 (4 to 8)	31·6% (16·4 to 47·4)	127 (100 to 157)	24·5% (18·2 to 31·0)	78 (59 to 101)	30·3% (16·0 to 44·6)
Chad	51 (38 to 67)	11·3% (−0·6 to 24·3)	1284 (1005 to 1642)	12·0% (6·4 to 18·3)	839 (635 to 1087)	11·3% (−0·4 to 24·6)
Côte d'Ivoire	110 (81 to 144)	20·8% (8·7 to 35·4)	2747 (2142 to 3475)	13·7% (8·5 to 19·8)	1923 (1440 to 2501)	20·4% (9·0 to 34·6)
The Gambia	8 (6 to 10)	18·2% (3·2 to 35·1)	186 (146 to 237)	9·6% (3·8 to 15·8)	126 (96 to 162)	17·0% (3·2 to 32·0)
Ghana	171 (126 to 225)	28·4% (12·5 to 50·3)	4048 (3183 to 5118)	13·5% (7·8 to 19·4)	2768 (2085 to 3568)	26·5% (11·5 to 45·2)
Guinea	58 (43 to 78)	17·9% (3·1 to 33·5)	1441 (1121 to 1836)	11·0% (5·8 to 16·8)	993 (733 to 1315)	17·5% (2·8 to 32·3)
Guinea-Bissau	8 (6 to 11)	16·2% (2·1 to 33·4)	217 (169 to 274)	13·1% (8·0 to 18·8)	148 (111 to 191)	15·5% (2·7 to 30·5)
Liberia	19 (14 to 25)	17·6% (4·5 to 32·5)	508 (400 to 648)	10·6% (5·2 to 16·6)	332 (249 to 427)	17·1% (5·6 to 30·8)
Mali	65 (47 to 86)	23·1% (6·3 to 44·5)	1567 (1224 to 2024)	13·2% (7·4 to 18·9)	1049 (767 to 1395)	20·0% (4·4 to 38·7)
Mauritania	23 (17 to 31)	20·5% (0·3 to 42·0)	565 (441 to 708)	15·7% (9·5 to 21·7)	381 (278 to 501)	18·9% (0·4 to 38·8)
Niger	62 (45 to 82)	14·0% (−1·4 to 34·1)	1732 (1351 to 2224)	8·0% (2·4 to 14·5)	1105 (809 to 1450)	13·1% (−1·6 to 31·5)
Nigeria	613 (429 to 835)	27·8% (6·4 to 48·8)	20 851 (16 306 to 26 258)	19·8% (13·4 to 26·6)	11 302 (8080 to 15 109)	26·5% (7·3 to 45·9)
São Tomé and Príncipe	1 (1 to 2)	22·2% (4·1 to 44·0)	27 (22 to 34)	13·8% (8·2 to 20·1)	17 (13 to 23)	19·5% (2·9 to 38·4)
Senegal	80 (60 to 105)	24·8% (13·2 to 39·5)	1688 (1322 to 2154)	11·2% (5·5 to 17·1)	1274 (965 to 1646)	22·2% (11·4 to 35·5)
Sierra Leone	20 (15 to 26)	15·4% (2·2 to 31·0)	637 (498 to 814)	12·7% (6·4 to 18·7)	384 (286 to 496)	15·6% (2·7 to 29·6)
Togo	31 (23 to 40)	17·5% (4·3 to 33·1)	794 (619 to 1007)	15·1% (8·8 to 21·6)	538 (403 to 695)	17·9% (5·0 to 32·3)
**Eastern sub-Saharan Africa**	**1975 (1493 to 2591)**	**34·6% (25·8 to 44·6)**	**46 489 (36 657 to 57 613)**	**21·7% (18·6 to 25·0)**	**30 752 (23 631 to 39 371)**	**31·5% (24·0 to 40·0)**
Burundi	42 (30 to 57)	25·4% (8·5 to 43·7)	1115 (880 to 1407)	15·9% (9·4 to 23·1)	683 (511 to 896)	23·0% (7·5 to 41·0)
Comoros	4 (3 to 5)	31·0% (14·4 to 48·5)	101 (80 to 126)	23·0% (16·6 to 29·8)	65 (50 to 84)	29·1% (14·5 to 45·8)
Djibouti	6 (5 to 9)	38·2% (14·5 to 63·0)	153 (119 to 191)	27·3% (20·3 to 35·4)	101 (74 to 135)	35·6% (13·5 to 57·3)
Eritrea	19 (14 to 26)	43·7% (26·1 to 63·1)	531 (419 to 668)	26·9% (19·7 to 34·1)	344 (256 to 442)	39·7% (24·6 to 57·2)
Ethiopia	556 (405 to 750)	40·7% (21·9 to 63·6)	12 384 (9654 to 15 735)	24·9% (17·8 to 32·4)	8580 (6437 to 11 338)	36·9% (19·5 to 56·8)
Kenya	232 (169 to 309)	40·9% (23·6 to 67·9)	6557 (5228 to 8065)	22·0% (19·7 to 24·7)	3714 (2782 to 4800)	36·4% (22·8 to 54·2)
Madagascar	99 (73 to 134)	17·2% (0·3 to 34·9)	2940 (2333 to 3665)	14·1% (7·6 to 20·2)	1657 (1221 to 2127)	15·9% (0·2 to 32·0)
Malawi	97 (69 to 130)	28·3% (3·4 to 60·4)	2112 (1650 to 2683)	19·0% (11·6 to 25·6)	1497 (1091 to 1973)	26·9% (3·1 to 56·7)
Mozambique	157 (114 to 212)	21·0% (4·1 to 42·4)	3376 (2626 to 4246)	20·7% (13·1 to 27·9)	2420 (1783 to 3224)	20·7% (4·6 to 40·7)
Rwanda	61 (44 to 81)	52·5% (32·3 to 74·8)	1376 (1089 to 1714)	24·6% (17·9 to 31·6)	903 (660 to 1176)	45·8% (27·7 to 68·3)
Somalia	42 (31 to 57)	19·5% (4·9 to 37·2)	1107 (865 to 1405)	13·8% (7·4 to 19·7)	700 (517 to 919)	17·8% (4·3 to 34·4)
South Sudan	52 (37 to 71)	34·2% (13·5 to 58·2)	1421 (1115 to 1789)	15·9% (9·2 to 23·1)	852 (616 to 1125)	30·5% (11·5 to 51·7)
Tanzania	353 (266 to 460)	35·2% (17·6 to 56·2)	7443 (6048 to 9048)	24·0% (13·4 to 34·4)	5302 (4081 to 6708)	31·5% (15·5 to 51·4)
Uganda	167 (123 to 226)	29·6% (13·2 to 49·0)	3873 (3046 to 4804)	23·9% (17·1 to 30·6)	2553 (1929 to 3310)	28·6% (13·3 to 46·5)
Zambia	88 (63 to 119)	33·8% (6·1 to 70·2)	1963 (1538 to 2453)	20·4% (14·2 to 27·3)	1376 (1009 to 1844)	33·1% (6·5 to 66·0)
**Central sub-Saharan Africa**	**474 (346 to 621)**	**22·0% (12·7 to 33·8)**	**13 564 (10 608 to 17 192)**	**10·1% (5·9 to 14·5)**	**8083 (6104 to 10 365)**	**19·4% (11·3 to 29·8)**
Angola	88 (62 to 123)	50·2% (21·6 to 89·2)	2615 (2034 to 3271)	19·7% (13·2 to 26·4)	1555 (1125 to 2107)	41·8% (16·8 to 74·1)
Central African Republic	24 (17 to 32)	10·9% (−4·9 to 26·3)	717 (559 to 915)	8·8% (2·7 to 14·9)	401 (293 to 525)	10·5% (−3·8 to 25·4)
Congo (Brazzaville)	30 (21 to 41)	23·6% (3·5 to 45·1)	789 (623 to 986)	16·4% (9·7 to 23·5)	490 (361 to 648)	21·5% (2·7 to 42·5)
Democratic Republic of the Congo	306 (224 to 404)	16·0% (4·8 to 30·1)	8845 (6920 to 11 285)	6·7% (1·0 to 12·2)	5244 (3904 to 6686)	14·4% (4·8 to 26·2)
Equatorial Guinea	5 (3 to 8)	58·3% (11·8 to 108·9)	159 (126 to 197)	42·3% (33·8 to 50·8)	91 (59 to 131)	52·4% (12·3 to 97·9)
Gabon	21 (15 to 28)	28·3% (9·4 to 51·0)	439 (346 to 550)	18·1% (12·4 to 24·8)	302 (223 to 395)	25·8% (7·9 to 46·6)

95% uncertainty intervals are in parentheses. DALYs=disability-adjusted life-years. SDI=Socio-demographic Index. For more details about the rationale for this classification of countries see reference 23.

Globally, Parkinson's disease caused 211 296 deaths (95% UI 167 771–265 160; 93 512, 95% UI 73 702–118 421, in women and 117 784, 93 729–147 607, in men) and 3·2 million DALYs (95% UI 2·6–4·0; 1·4 million, 95% UI 1·1–1·7, in women; 1·8 million, 1·5–2·3, in men) in 2016. Of these, high SDI countries accounted for 84 911 (40·2%) deaths and 1·1 million (34·4%) DALYs, high-middle or middle SDI countries for 98 820 (46·8%) deaths and 1·6 million (50·0%) DALYs, and low-middle or low SDI countries for 27 470 (13·0%) deaths and 0·5 million (15·6%) DALYs. The number of deaths was 2·6 times higher and the number of DALYs was 2·5 times higher in 2016 than in 1990. These increases were not explained exclusively by an increasing number of older people, because age-standardised rates increased from 1990 to 2016 for both deaths and DALYs by about 20% (table). Similar to prevalence, the increases in deaths and DALYs were lowest in high SDI countries and highest in middle SDI countries, and were seen in both men and women.

**Figure 2:**
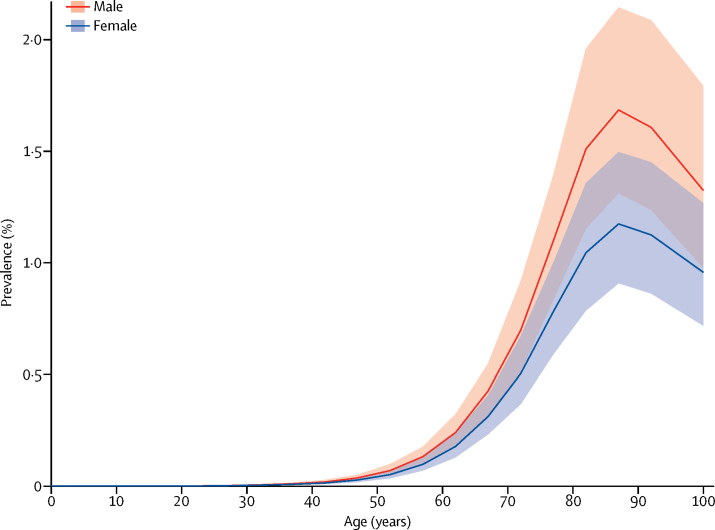
Global prevalence of Parkinson's disease by age and sex, 2016 Prevalence is expressed as the percentage of the population that is affected by the disease. Shading indicates 95% uncertainty intervals.

**Figure 3:**
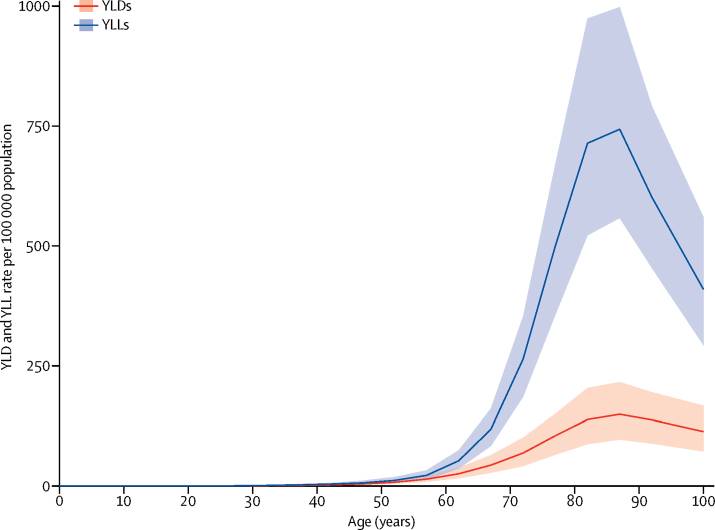
Global YLD and YLL rates per 100 000 population due to Parkinson's disease by age, 2016 Shading indicates 95% uncertainty intervals. YLDs=years lived with disability. YLLs=years of life lost.

Parkinson's disease was uncommon before 50 years of age. Prevalence in 2016 increased with age thereafter and peaked between 85 years and 89 years (1·7% for men; 1·2% for women) and decreased after that age (figure 2). Age-standardised prevalence of Parkinson's disease in 2016 was 1·40 times (95% UI 1·36–1·43) higher in men than in women; the male-to-female ratio was similar in 1990 (1·37, 95% UI 1·34–1·40). A similar pattern was seen for the rates of YLLs and YLDs according to age, although the age-related increase was considerably steeper for YLLs than for YLDs, suggesting that Parkinson's disease-related case fatality rises with age (figure 3).

**Figure 4:**
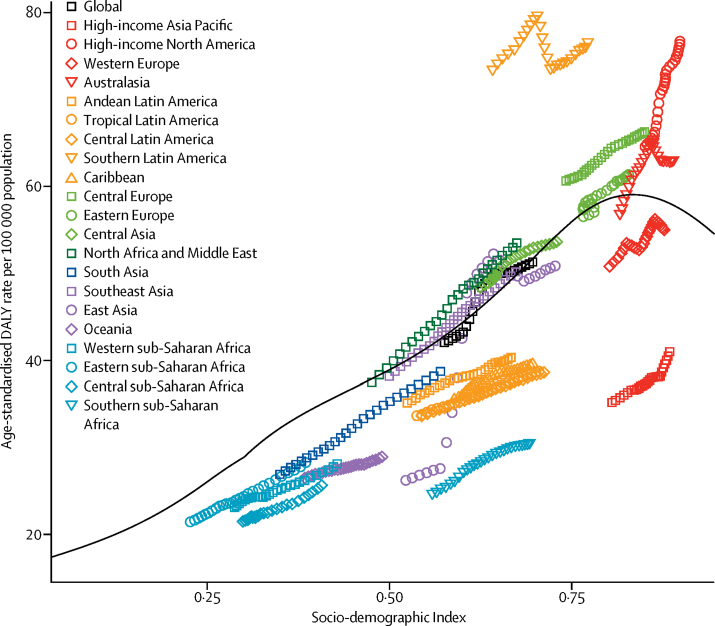
Age-standardised DALY rates for Parkinson's disease by 21 Global Burden of Disease regions by Socio-demographic Index, 1990–2016 Expected values based on Socio-demographic Index and disease rates in all locations are shown as the black line. The black line represents expected values of age-standardised DALY rates for each value of Socio-demographic Index and is based on a Gaussian process regression of results for all Global Burden of Disease locations over the entire 1990–2016 estimation period. DALY=disability-adjusted life-year.

The age-standardised rate of DALYs of Parkinson's disease by the 21 GBD world regions generally increased with SDI (figure 4). Sub-Saharan Africa, Latin America and the Caribbean (with the exception of southern Latin America), south Asia, and high-income Asia Pacific had lower age-standardised DALY rates than other regions with similar SDI. Southern Latin America and high-income North America were the regions with highest estimates relative to expected values based on SDI.

Smoking was found to have a small, protective effect on Parkinson's disease, and would have been expected to prevent 461 194 DALYs (95% UI 324 745–599 845) globally in 2016 if the association was truly causal.

## Discussion

Over the past generation, the number of individuals with Parkinson's disease globally has more than doubled to over 6 million. Of all the neurological disorders included in GBD 2015,^1^ Parkinson's disease was the fastest growing. Ageing populations contributed to much of that growth as crude prevalence rates increased by about 74% from 1990 to 2016 and age-standardised prevalence rates increased by about 22%. However, because age-standardised prevalence, DALYs, and death rates all increased from 1990 to 2016, additional factors are probably important.

First, changes in study methods, availability of higher-quality studies, and greater awareness of diagnosis^24^ might have led to better estimates of prevalence, DALYs, and deaths since 1990.^3^ For example, door-to-door studies are less likely to miss individuals who have never been diagnosed and would be missed in health records.^25^ Our DisMod-MR 2.1 model did not show evidence in favour of a systematic bias between door-to-door surveys and studies based on administrative records; however, establishing such evidence in a model with relatively sparse data is difficult. Although many regions have seen improvements in study methods, these changes alone are probably insufficient to explain the rising burden of Parkinson's disease. For example, the rates of Parkinson's disease have also increased in high-income countries without substantial changes in study methodology.

Second, increasing life expectancy is probably contributing to longer disease duration in individuals with Parkinson's disease and thus to higher prevalence, even if incidence remains constant and individuals with Parkinson's disease show the same time trends in mortality as the general population.^12^ Indeed, in a meta-analysis^26^ of ten studies, recent cohorts showed longer disease duration, with an increase of 2·5 years per decade. That study^26^ showed no clear evidence that the introduction of levodopa and improvements in Parkinson's disease care have led to improvement in survival of individuals with Parkinson's disease compared with similar individuals without Parkinson's disease. A recent study^12^ estimated the burden of Parkinson's disease in France from 2010 to 2030 under a constant incidence scenario and assuming that the relative risk of death of individuals with Parkinson's disease relative to controls had not changed over time. It showed that the life expectancy of individuals with Parkinson's disease would be expected to increase by approximately 3 years and the age-standardised and sex-standardised prevalence rate by 12% over 20 years. As patients live with Parkinson's disease for more years and the number of individuals with advanced Parkinson's disease increases, studies will be needed to inform the distribution of the severity of the disease in representative samples with simple instruments such as the Hoehn and Yahr scale.

Third, the increase in Parkinson's disease burden might be linked to environmental factors tied to the growing industrialisation of the world. In general, better health is positively associated with socioeconomic level.^27^, ^28^, ^29^ However, with Parkinson's disease, the opposite is true; age-standardised DALY rates due to Parkinson's disease increased with SDI. The reason for this association is not clear. Some environmental exposures tied to industrialisation, including pesticides,^7^ solvents,^7^ or metals,^8^, ^9^ which are also more common in high SDI countries, might contribute to the increased incidence of Parkinson's disease. For example, in China (a middle SDI country), which has undergone rapid industrial growth since 1990, the age-adjusted prevalence rates of Parkinson's disease more than doubled between 1990 and 2016, the largest increase worldwide. If environmental factors related to industrialisation played a part, an increase in incidence over time would be expected. A few studies have examined time trends in incidence of Parkinson's disease, with inconsistent findings. A study in the USA^30^ suggested that incidence increased by 24% (95% CI 8–43) per decade between 1976 and 2005 in men but not in women. By contrast, in the Netherlands, the Rotterdam Study^31^ reported a substantial decrease in Parkinson's disease incidence between 1990 and 2011, without any obvious explanation. One study in Canada^32^ and another in the USA^33^ study showed no significant time trends. In Finland, using the Finnish National Prescription Register, the incidence of Parkinson's disease increased between 1997 and 2014 both in rural and urban regions.^34^ High-quality prospective cohort studies and detailed registries are needed to survey time trends in the worldwide incidence of Parkinson's disease more accurately and understand the factors that might be driving time trends. Alternative explanations for the positive association between the burden of Parkinson's disease and SDI include better ascertainment of Parkinson's disease in higher SDI countries through better study methods or health-care access and disease recognition. However, in lower SDI countries, we included door-to-door studies, when available, that are considered to be less prone to underestimation.^25^

Fourth, declining smoking rates in some countries,^35^ although a global health boon, might contribute to higher incidence of Parkinson's disease.^14^ The risk of Parkinson's disease is decreased by approximately 40% among smokers.^10^ Whether this association is truly causal or explained by reverse causation or other biases is still debated.^11^ If the association between smoking and Parkinson's disease were causal, decreasing smoking rates would lead to an increase in the incidence of Parkinson's disease in the future. Assuming a causal inverse association and a 10-year lag to account for the temporal effect of smoking on the incidence of Parkinson's disease, one study in the USA^14^ estimated that declining smoking rates in the country might increase the projected burden of Parkinson's disease in 2040 by 10%. However, because the lag time between exposure and the actual effect on disease risk is unknown and might actually be longer than 10 years,^36^ the timing of the potential effect of declining smoking rates on Parkinson's disease incidence is uncertain, and additional studies are needed to examine the effects of changing smoking habits in different parts of the world with different smoking rates and time trends. Regardless of the results of such studies, the adverse health consequences of smoking far outweigh any potential benefit on Parkinson's disease. Finally, changes in the prevalence of other known (eg, head trauma^37^) or unknown risk or protective factors that were not included in GBD 2016 might contribute to changing incidence rates of Parkinson's disease.

This study confirms that Parkinson's disease is about 1·4 times more frequent in men than women, and this ratio did not change substantially over the study period. Environmental (eg, occupational) exposures to which men are more frequently exposed might contribute to this pattern. The prevalence of Parkinson's disease increased with age. Underascertainment at older ages owing to underdiagnosis, comorbidities, or institutional care might explain the decrease seen in the oldest age groups after the peak between 85 years and 89 years.

The current estimates for the global burden of Parkinson's disease are generated from imperfect data and models that are refined in each iteration of the GBD study. Estimates from GBD studies can vary from year to year as revised estimates are generated on the basis of refined methods and inclusion of more and higher-quality studies that are less likely to underestimate the true burden of the disease. Nonetheless, high-quality epidemiological studies (especially on incidence and disease severity) are still rare for large portions of the world, especially in low-income regions, where such studies are needed to understand trends and guide efforts to reduce the disease burden. Methodological differences for determining prevalence and study shortcomings might result in estimates that vary considerably and underestimate the true burden of Parkinson's disease.^38^ This under-reporting is well known for Parkinson's disease in studies based on death certificates,^39^, ^40^, ^41^, ^42^, ^43^ whereas population-based door-to-door studies are considered a better approach because they are able to capture undiagnosed cases.^25^ However, disease frequency estimates from population-based studies might be affected by selection bias resulting from non-response, particularly if individuals affected by the disease under investigation are less likely to participate. Non-response is an important issue as participation rates in epidemiological studies have considerably decreased over the past 30 years.^44^ Another limitation is that because we lack strong predictors for the occurrence of Parkinson's disease, some of the variation between countries is probably due to measurement error that we have been unable to correct. Because we rely on prevalence data to derive our cause of death estimates, any residual measurement error in the prevalence estimates is transposed onto the death estimates for Parkinson's disease. Although the the bias in mortality is an unwanted property, it is less than would have been the case if we had based our estimates on the observed rates of death with Parkinson's disease as the underlying cause from vital registration data. The large variation in death rates over time within the same countries and the even larger variation between countries are implausible and probably explained by changing death coding practices.

Neurological disorders are now the leading source of disability in the world, and Parkinson's disease is the fastest growing of these disorders.^1^ As the population ages and life expectancy increases, the doubling of the number of individuals with Parkinson's disease between 1990 and 2016 is projected to occur again in the coming generation.^12^, ^13^, ^14^, ^45^ To address this great health challenge will require action aimed at preventing the disease where feasible and improving the lives of those affected by the condition.^46^ Among the potential responses available are preventing the disease (eg, by increasing physical activity earlier in adulthood^47^ and reducing exposure to pesticides^7^), improving worldwide access to care and effective treatments (eg, levodopa), increasing funding for research (eg, to understand the underlying causes), and development of new therapies.
