# Correction to *Lancet Neurol* 2018; 17: 939–53

**DOI:** 10.1016/S1474-4422(21)00382-3

**Published:** 2021-12

*GBD 2016 Parkinson's Disease Collaborators. Global, regional, and national burden of Parkinson's disease, 1990–2016: a systematic analysis for the Global Burden of Disease Study 2016.* Lancet Neurol *2018; **17:** 939–53*—In this Article, Nooshin Abbasi and Samer Hamidi should have been included in the GBD 2016 Parkinson's Disease Collaborators. The affiliations have also been updated. These corrections have been made to the online version as of Nov 17, 2021.

