# Supplementary material for: Global, regional, and national burden of Parkinson's disease, 1990–2016: a systematic analysis for the Global Burden of Disease Study 2016
Source: Lancet Neurol. 2018 Nov;17(11):939–53. doi: 10.1016/S1474-4422(18)30295-3 (PMC6191528; doi:10.1016/S1474-4422(18)30295-3)
Supplement: Supplementary appendix [file mmc1.pdf]

THE LANCET
Neurology

Supplementary appendix

This appendix formed part of the original submission and has been peer reviewed. We post it as supplied by the authors.

Supplement to: GBD 2016 Parkinson's Disease Collaborators. Global, regional, and national burden of Parkinson's disease, 1990–2016: a systematic analysis for the Global Burden of Disease Study 2016. *Lancet Neurol* 2018; published online Oct 1. http://dx.doi.org/10.1016/S1474-4422(18)30295-3.

Appendix 1

Summary of General Global Burden of Disease Study Methods

The Institute of Health Metrics and Evaluation with a growing collaboration of scientists produces annual updates of the Global Burden of Disease study. Estimates span the period from 1990 to the most recent completed year. By the time of the release of GBD 2016 in September 2017, there were over 2,700 collaborators in 132 countries who contributed to this global public good. Annual updates allow incorporation of new data and method improvements to ensure that the most up-to-date information is available to policy makers in a timely fashion to help make resource allocation decisions. In this analysis, we have aggregated results from GBD 2016 for 15 disease and injury outcomes that are generally cared for by neurological services. These include infectious conditions (tetanus, meningitis, encephalitis), stroke, brain and other nervous system cancers, traumatic brain injury and spinal cord lesion which are classified outside the more narrowly defined category of neurological disorders in GBD (i.e., Alzheimer disease and other dementias, Parkinson disease, multiple sclerosis, motor neuron disease, idiopathic epilepsy, migraine, tension-type headache and a rest category of less common other neurological disorders). Compared to a previous analysis based on GBD 2015,[1] we were able to add the non-fatal outcomes of traumatic brain injury and spinal cord lesion and medication overuse headache is no longer included as a separate cause but quantified as a consequence of the underlying headache types.

In the methods section of this overview paper we present a summary of the general methods of the global burden of disease. In the accompanying disease-specific papers we concentrate on methods that are specific to each disorder. The guiding principle of GBD is to assess health loss due to mortality and disability comprehensively where we define disability as any departure from full health. In GBD2016, estimates were made for 195 countries and territories, and 579 subnational locations, for 27 years starting from 1990, for 23 age groups and both sexes. Deaths were estimated for 264 disease and injuries while prevalence and incidence were estimated for 328 diseases and injuries. In order to allow meaningful comparisons between deaths and non-fatal disease outcomes as well as between diseases, the data on deaths and prevalence are summarized in a single indicator, the disability-adjusted life year (DALY). DALYs are the sum of years of life lost (YLLs) and years lived with disability (YLDs). YLLs are estimated as the multiplication of counts of death and a standard, 'ideal', remaining life expectancy at the age of death. The standard life expectancy is derived from the lowest observed mortality rates in any population in the world greater than 5 million.[2] YLDs are estimated as the product of prevalence of individual consequences of disease (or 'sequelae') times a disability weight that quantifies the relative severity of a sequela as a number between zero (representing 'full health') and 1 (representing death). Disability weights have been estimated in nine population surveys and an open-access internet survey in which respondents are asked to choose the 'healthier'[3] between random pairs of health states that are presented with a short description of the main features.

All-cause mortality rates are estimated from vital registration data in countries with complete coverage. For other countries, the probabilities of death before age 5 and between ages 15 and 60 are estimated from censuses and surveys asking mothers to provide a history of children ever born and those still alive, and surveys asking adults about siblings who are alive or have passed away. Using model life tables, these probabilities of death are transformed into age-specific death rates by location,

year and sex. GBD has collated a large database of cause of death data from vital registrations and verbal autopsy surveys in which relatives are asked a standard set of questions to ascertain the likely cause of death, supplemented with police and mortuary data for injury deaths in countries with no other data. For countries with vital registration data, the completeness is assessed with demographic methods based on comparing recorded deaths with population counts between two successive censuses. The cause of death information is provided in a large number of different classification systems based on versions of the International Classification of Diseases or bespoke classifications in some countries. All data are mapped into the disease and injury categories of GBD. All classification systems contain codes that are less informative because they lack a specific diagnosis (e.g. unspecified cancer) or refer to codes that cannot be underlying cause of death (e.g., low back pain or senility) or are intermediate causes (e.g., heart failure or sepsis). Such deaths are redistributed to more precise underlying causes of death.[4] After these redistributions and corrections for under-registration the data are analysed in CODEm (cause of death ensemble model), a highly systematized tool that runs many different models on the same data and chooses an ensemble of models that best reflects all the available input data. Models are chosen with variations in the statistical approach ('mixed effects' of space-time Gaussian Process Regression), in the unit of analysis (rates or cause fractions), and the choice of predictive covariates. The statistical performance of all models is tested by holding out 30% of the data and checking how well a model covers the data that were held out. To enforce consistency from CODEm, the sum of all cause-specific mortality rates is scaled to that of the all-cause mortality rates in each age, sex, location and year category.

Non-fatal estimates are based on systematic reviews of published papers and unpublished documents, survey microdata, administrative records of health encounters, registries and disease surveillance systems. Our Global Health Data Exchange (GHDx, http://ghdx.healthdata.org/) is the largest repository of health data globally. We first set a reference case definition and/or study method that best quantifies each disease or injury or consequence thereof. If there is evidence of a systematic bias in data that used different case definitions or methods compared to reference data we adjust those data points to reflect what its value would have been if measured as the reference. This is a necessary step if one wants to use all data pertaining to a particular quantity of interest rather than choosing a small subset of data of the highest quality only. DisMod-MR 2.1, a Bayesian meta-regression tool, is our main method of analyzing non-fatal data. It is designed as a geographical cascade where a first model is run on all the world's data which produces an initial global fit and estimates coefficients for predictor variables and the adjustments for alternative study characteristics. The global fit adjusted by the values of random effects for each of 7 GBD super-regions, the coefficients on sex and country predictors, are passed down as data to a model for each super-region together with the input data for that geography. The same steps are repeated going from super-region to 21 region fits and then to 195 fits by country and where applicable a further level down to subnational units. Below the global fit, all models are run separately by sex and for 6 time periods: 1990, 1995, 2000, 2005, 2010 and 2016. During each fit all data on prevalence, incidence, remission (i.e., cure rate) and mortality are forced to be internally consistent. For most diseases, the bulk of data on prevalence or incidence is at the disease level with fewer studies providing data on the proportions of cases of disease in each of the sequelae defined for the disease. The proportions in each sequela are pooled using DisMod-MR 2.1 or meta-analysis, or derived from analyses of patient-level data sets. The multiplication of prevalent cases for each disease sequela and the appropriate disability weight produces YLD estimates that do not yet take into account comorbidity. To correct for comorbidity, these data are used in a simulation to create hypothetical individuals in each age, sex, location and year combination who experience no, one or multiple sequelae simultaneously. We assume that disability weights are multiplicative rather than

additive as this avoids assigning a combined disability weight value in any individual to exceed 1, i.e., be worse than a 'year lost due to death'. This comorbidity adjustment leads to an average scaling down of disease-specific YLDs ranging from around 2% in young children up to 17% in oldest ages.

All our estimates of causes of death are categorical: each death is assigned to a single underlying cause. This has the attractive property that all estimates add to 100%. For risks, we use a different, 'counterfactual', approach, i.e. answering the question: "what would the burden have been if the population had been exposed to a theoretical minimum level of exposure to a risk". Thus, we need to define what level of exposure to a risk factor leads to the lowest amount of disease. We then analyse data on the prevalence of exposure to a risk and derive relative risks for any risk-outcome pair for which we find sufficient evidence of a causal relationship. Prevalence of exposure is estimated in DisMod-MR 2.1, using space-time Gaussian Process Regression, or from satellite imagery in the case of ambient air pollution. Relative risk data are pooled using meta-analysis of cohort, case-control and or intervention studies. For each risk and outcome pair, we evaluate the evidence and judge if the evidence falls into the categories of 'convincing' or 'probable' as defined by the World Cancer Research Fund.[5] From the prevalence and relative risk results, population attributable fractions are estimated relative to the theoretical minimum risk exposure level (TMREL). When we aggregate estimates for clusters of risks, e.g. metabolic or behavioral risks, we use a multiplicative function rather than simple addition and take into account how much of each risk is mediated through another risk. For instance, some of the risk of high body mass index is directly onto stroke as an outcome but much of its impact if mediated through high blood pressure, high cholesterol or high fasting plasma glucose and we would not want to double count the mediated effects when we estimate aggregates across risk factors.[6]

Uncertainty is propagated throughout all these calculations by creating 1,000 values for each prevalence, death, YLL, YLD or DALY estimate and performing aggregations across causes and locations at the level of each of the 1,000 values for all intermediate steps in the calculation. The lower and upper bounds of the 95% uncertainty interval are the 25th and 975th values of the ordered 1,000 values. For all age-standardised rates, GBD uses a standard population calculated as the non-weighted average across all countries of the percentage of the population in each 5-year age group for the years 2010 to 2035 from the United Nations Population Division's World Population Prospects (2012 revision).[7,8]

GBD uses a composite indicator or sociodemographic development, SDI, which reflects the geometric mean of normalized values of a location's income per capita, the average years of schooling in the population 15 and over, and the total fertility rate. Countries and territories are grouped into 5 quintiles of high, high-middle, middle, low-middle, and low SDI based on their 2016 values.[2]

1 GBD 2015 Neurological Disorders Collaborator Group. Global, regional, and national burden of neurological disorders during 1990-2015: a systematic analysis for the Global Burden of Disease Study 2015. *Lancet Neurol* 2017; **16**: 877–97.

2 GBD 2016 Mortality Collaborators. Global, regional, and national under-5 mortality, adult mortality, age-specific mortality, and life expectancy, 1970-2016: a systematic analysis for the Global Burden of Disease Study 2016. *Lancet Lond Engl* 2017; **390**: 1084–150.

3 Salomon JA, Haagsma JA, Davis A, *et al.* Disability weights for the Global Burden of Disease 2013 study. *Lancet Glob Health* 2015; **3**: e712-723.

4 GBD 2016 Causes of Death Collaborators. Global, regional, and national age-sex specific mortality for 264 causes of death, 1980-2016: a systematic analysis for the Global Burden of Disease Study 2016. *Lancet Lond Engl* 2017; **390**: 1151–210.

5 American Institute for Cancer Research. Food, nutrition, physical activity, and the prevention of cancer: a global perspective. Washington, DC: American Institute for Cancer Research, 2007.

6 GBD 2016 Risk Factors Collaborators. Global, regional, and national comparative risk assessment of 84 behavioural, environmental and occupational, and metabolic risks or clusters of risks, 1990-2016: a systematic analysis for the Global Burden of Disease Study 2016. *Lancet Lond Engl* 2017; **390**: 1345–422.

7 GBD 2013 Mortality and Causes of Death Collaborators. Global, regional, and national age-sex specific all-cause and cause-specific mortality for 240 causes of death, 1990-2013: a systematic analysis for the Global Burden of Disease Study 2013. *Lancet Lond Engl* 2015; **385**: 117–71.

8 United Nations Department of Economics and Social Affairs Population Division. World Population Prospects: The 2012 Revision. http://esa.un.org/unpd/wpp/Documentation/publications.htm (accessed Nov 4, 2014).

Appendix 2

Parkinson's Disease (Nonfatal)

Flowchart

Parkinson's Disease

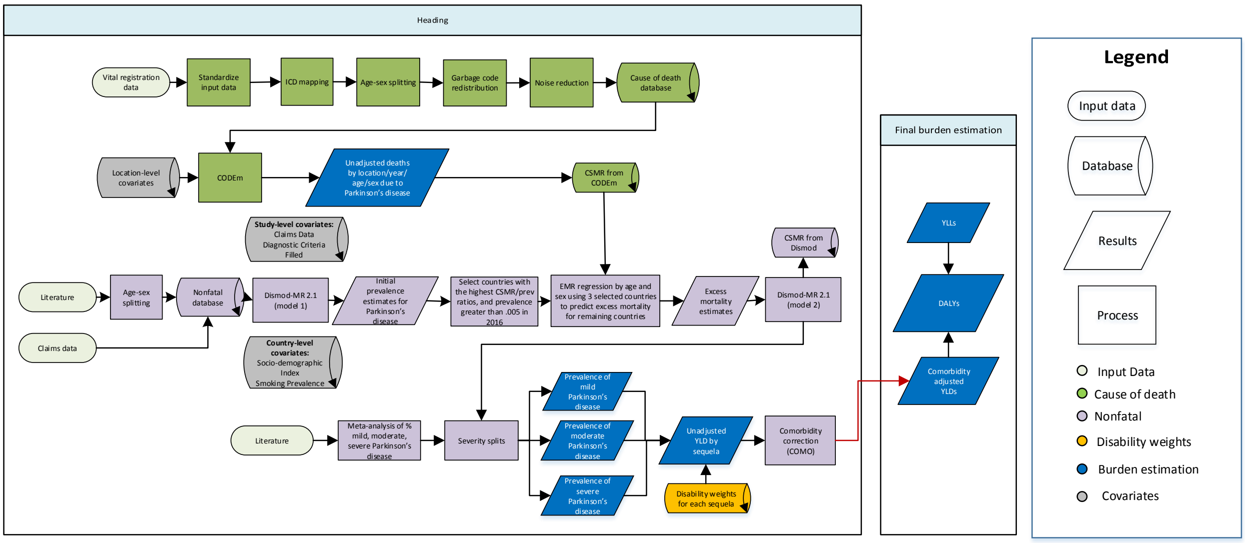

Case definition

Parkinson's disease is a chronic, degenerative, and progressive neurological condition typified by the loss of motor mobility and control – most notably tremors. The corresponding ICD-10 codes are G20, G21, and G22. Our case definition for GBD is the presence of at least two of the four primary symptoms: (1) tremors/trembling, (2) bradykinesia, (3) stiffness of limbs and torso, and (4) posture instability.

Unlike most causes in the Global Burden of Disease project, Parkinson's disease mortality and morbidity estimates are modelled jointly. This is because of marked discrepancies between prevalence data and cause of death data. Specifically, prevalence data suggest little to no variation over time (eg, 1990–2016) whereas age-standardised mortality rates in vital registrations in high-income countries have increased multiple times over this same period. Additionally, prevalence variation between countries is much smaller than the variation in death rates assigned to Parkinson's disease in vital registration. We attribute these discrepancies to changing coding practices rather than epidemiological change.

Because of this joint procedure, descriptions of the mortality estimation process are included where relevant.

Input data

Model inputs

To inform our estimates of burden due to Parkinson's disease, we use mortality data from vital registration systems, as well as prevalence data from surveys and administrative data such as claims sources.

For GBD 2015, the systematic review was updated using a search spanning from January 2011 to December 2015, and the search terms were set to capture studies for Parkinson's disease.[1] This search term resulted in 1,433 initial hits with 17 sources marked for extraction. Studies with no clearly defined sample or that drew from specific clinic/patient organizations were excluded.

The following table provides a description of the density and distribution of literature data informing the Parkinson's estimates:

	Prevalence	Incidence	Mortality risk
Studies	94	34	10
Regions	16	9	3

Beyond the exclusion of studies using non-representative populations, there are no substantial adjustment or outliering criteria for the Parkinson's model. Certain studies have been outliered on a case-by-case basis due to subsequent review and exclusion due to inappropriateness of the study design, and overly broad age and sex groups that conflict with existing gold-standard age-sex-specific data – where possible.

Severity splits

As in GBD 2013, we use Hoehn and Yahr stages to determine severity. However, for GBD 2016, the cutpoints were updated in order to more accurately correspond with the lay descriptions of severities. Specifically, a Hoehn and Yahr stage 4 now corresponds to a designation of severe, where before it was classified as moderate.

Severity	Stage
Mild	≤2.0
Moderate	2.5–3.0
Severe	≥4

For GBD 2016, a new literature review was completed to update the severity splits meta-analysis. The systematic review covered 1/1/2008 to 11/10/2016 and the search terms were set to capture studies reporting prevalence of Parkinson's by Hoehn and Yahr stage.[2] The search term resulted in 234 hits with 21 marked for extraction.

The following figures show the results of the meta-analysis on Hoehn and Yahr stage.

[1] (((Parkinson disease AND epidemiology) AND ("2011/01/01"[PDat]: "2015/12/31"[PDat])) AND (Parkinson disease AND epidemiology))

[2] (("1/1/2008"[Date - Publication] : "2016"[Date - Publication])) AND (parkinson disease[MeSH Terms] OR parkinson disease) AND (epidemiology OR prevalence OR incidence) AND (Hoehn) AND (Yahr) AND (stage)

Figure 1. Percentage of mild cases of Parkinson's disease in population-based studies

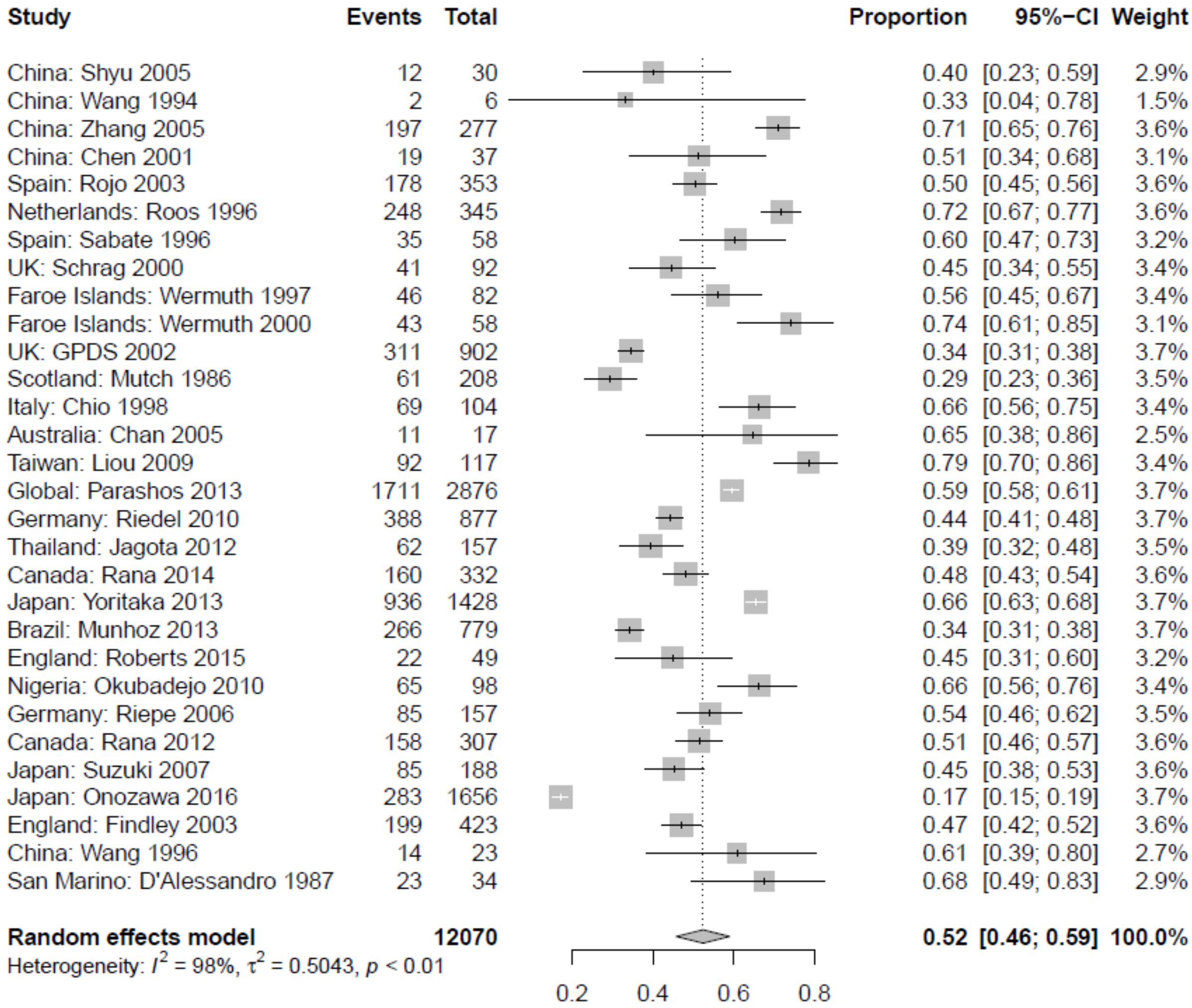

Figure 2. Percentage of moderate cases of Parkinson's disease in population-based studies

Study	Events	Total	Proportion	95%−CI	Weight
China: Shyu 2005	15	30	0.50	[0.31; 0.69]	2.7%
China: Wang 1994	2	6	0.33	[0.04; 0.78]	1.1%
China: Zhang 2005	51	277	0.18	[0.14; 0.23]	3.8%
China: Chen 2001	12	37	0.32	[0.18; 0.50]	2.8%
Spain: Rojo 2003	144	353	0.41	[0.36; 0.46]	4.0%
Netherlands: Roos 1996	94	345	0.27	[0.23; 0.32]	3.9%
Spain: Sabate 1996	12	58	0.21	[0.11; 0.33]	3.0%
UK: Schrag 2000	45	92	0.49	[0.38; 0.60]	3.5%
Faroe Islands: Wermuth 1997	16	82	0.20	[0.12; 0.30]	3.2%
Faroe Islands: Wermuth 2000	3	58	0.05	[0.01; 0.14]	1.8%
UK: GPDS 2002	444	902	0.49	[0.46; 0.53]	4.1%
Scotland: Mutch 1986	52	208	0.25	[0.19; 0.31]	3.8%
Italy: Chio 1998	15	104	0.14	[0.08; 0.23]	3.2%
Australia: Chan 2005	5	17	0.29	[0.10; 0.56]	2.0%
Taiwan: Liou 2009	18	117	0.15	[0.09; 0.23]	3.3%
Global: Parashos 2013	929	2876	0.32	[0.31; 0.34]	4.1%
Germany: Riedel 2010	339	877	0.39	[0.35; 0.42]	4.1%
Thailand: Jagota 2012	84	157	0.54	[0.45; 0.61]	3.8%
Canada: Rana 2014	121	332	0.36	[0.31; 0.42]	3.9%
Japan: Yoritaka 2013	414	1428	0.29	[0.27; 0.31]	4.1%
Brazil: Munhoz 2013	379	779	0.49	[0.45; 0.52]	4.1%
England: Roberts 2015	23	49	0.47	[0.33; 0.62]	3.2%
Nigeria: Okubadejo 2010	29	98	0.30	[0.21; 0.40]	3.5%
Germany: Riepe 2006	55	157	0.35	[0.28; 0.43]	3.7%
Canada: Rana 2012	105	307	0.34	[0.29; 0.40]	3.9%
Japan: Suzuki 2007	89	188	0.47	[0.40; 0.55]	3.8%
Japan: Onozawa 2016	959	1656	0.58	[0.55; 0.60]	4.1%
England: Findley 2003	120	423	0.28	[0.24; 0.33]	4.0%
China: Wang 1996	3	23	0.13	[0.03; 0.34]	1.7%
San Marino: D'Alessandro 1987	4	34	0.12	[0.03; 0.27]	2.0%
**Random effects model**		**12070**	**0.32**	**[0.28; 0.37]**	**100.0%**

Heterogeneity: $I^2 = 96\%$, $\tau^2 = 0.2632$, $p < 0.01$

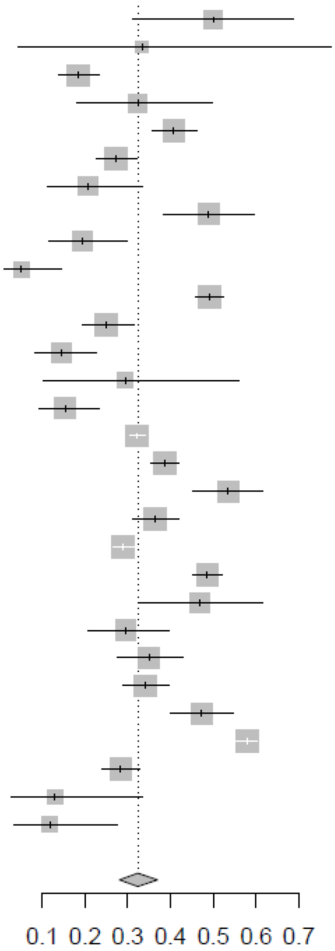

Figure 3. Percentage of severe cases of Parkinson's disease in population-based studies

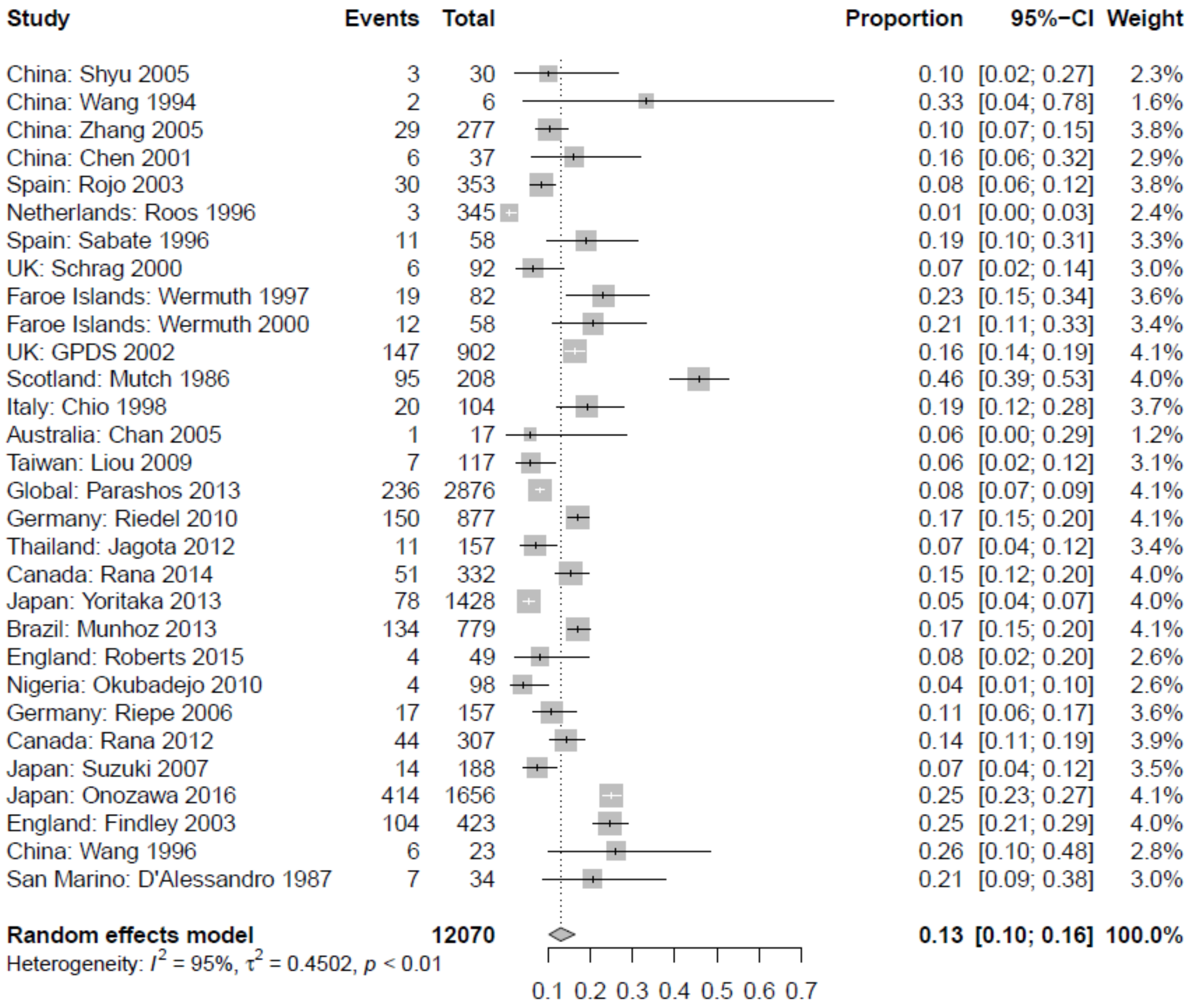

Severity estimates were generated by multiplying estimates of prevalence (country-year-sex-age-specific) by the fractions of mild, moderate, and severe PD and estimated 95% confidence intervals by taking 1,000 draws.

The following table provides the lay description and disability weights associated with Parkinson's disease.

Severity level	Lay description	DW (95% CI)
Mild	Has mild tremors and moves a little slowly, but is able to walk and do daily activities without assistance.	0.01 (0.005–0.019)
Moderate	Has moderate tremors and moves slowly, which causes some difficulty in walking and daily activities. The person has some trouble swallowing, talking, sleeping, and remembering things.	0.267 (0.181–0.372)
Severe	Has severe tremors and moves very slowly, which causes great difficulty in walking and daily activities. The person falls easily and has a lot of difficulty talking, swallowing, sleeping, and remembering things.	0.575 (0.396–0.73)

Modelling strategy

First, we ran a CODEm model for Parkinson's disease and extracted the mortality rates by age, sex, and geography for 2016.

Second, we ran a DisMod-MR 2.1 model with all data on incidence, prevalence, and mortality risk (relative risk, standardised mortality ratio, or with-condition mortality rates) and a setting of zero remission and extracted 2016 prevalence by age, sex, and geography. To account for potential systematic differences between claims and survey data, we crosswalked for each year of claims data.

Third, we selected the three countries (United States, Finland, and Austria) with the highest cause-specific mortality rate (from step 1) to prevalence (from step two) ratio, which also had prevalence rates above 0.0005 and a population greater than 1 million.

Fourth, we used a linear effects regression with dummies on age group and sex to predict excess mortality (EMR; ie, the ratio of cause-specific mortality rate and prevalence) by age and sex, the results of which are found in the tables below.

Fifth, these estimates were added to a second DisMod-MR 2.1 model as pertaining to the full 1990–2016 estimation period. For the three countries included in the regression, we retained their age- and sex-specific ratios and entered those also as pertaining to the full 1990–2016 estimation period. Thus, the model reflects the cause-specific mortality rate if all countries over time would have had the average propensity to code to Parkinson's disease as an underlying cause of death similar to the selected three countries in 2016.

Fixed-effect coefficients of EMR regression. Outcome: ln(EMR)					
Independent variables	Coef	Std.error	P value	95% confidence interval	
Male	0.214	0.074	0.006	0.069	0.359
Age 40-59	-3.522	0.157	0.000	-3.829	-3.214
Age 60-64	-2.716	0.157	0.000	-3.024	-2.409
Age 65-69	-2.236	0.157	0.000	-2.544	-1.929
Age 70-74	-1.686	0.157	0.000	-1.993	-1.378
Age 75- 80	-1.194	0.157	0.000	-1.502	-0.887
Age 80-84	-0.779	0.157	0.000	-1.087	-0.471
Age 85-89	-0.493	0.157	0.003	-0.800	-0.185
Age 90-94	-0.203	0.157	0.202	-0.511	0.104
Constant	-2.097	0.117	0.000	-2.326	-1.867

Predicted EMR values by age and sex (95% CI)		
	Male	Female
Age 40-59	0.005 (0.004–0.006)	0.004 (0.003–0.005)
Age 60-64	0.01 (0.008–0.013)	0.008 (0.006–0.01)
Age 65-69	0.016 (0.013–0.02)	0.013 (0.01–0.016)
Age 70-74	0.028 (0.023–0.035)	0.023 (0.018–0.029)
Age 75- 80	0.047 (0.037–0.059)	0.037 (0.029–0.046)
Age 80-84	0.071 (0.055–0.089)	0.057 (0.045–0.07)
Age 85-89	0.093 (0.073–0.117)	0.076 (0.06–0.093)
Age 90-94	0.126 (0.099–0.155)	0.101 (0.08–0.127)
Age 95+	0.154 (0.122–0.191)	0.123 (0.097–0.153)

In this model, we assumed zero remission among all ages, with no incidence or excess mortality for ages zero to 20 years old. We ignore data on incidence, relative risk, standardized mortality ratio, and with-condition mortality as these were shown to be inconsistent with prevalence estimates. We also constrain the super-region random effects for prevalence and incidence to -0.25 and 0.25 to account for spurious inflation of regional differences.

We make one study-level crosswalk: Diagnostic Criteria. Studies that do not use the gold-standard case definition of presence of at least two of the four main symptoms are crosswalked to meet this gold standard definition. The table below shows the effect of this crosswalk, which results in a downward adjustment of non-standard data points. For GBD 2015, an additional adjustment was made for Case Ascertainment, or studies that ascertain cases on clinical record review rather than using live diagnostic processes. However, this adjustment was not significant for GBD 2016 and was therefore changed to a z-cov, which affects the uncertainty of the estimates.

Additionally, claims data for 2000, 2010, and 2012 are adjusted via study covariates to account for systematic differences between the claims data and the literature. We use a country-level crosswalk to assist DisMod in estimating global patterns. We use Socio-demographic Index as a proxy to capture possible social and cultural risk factors or modifiers of Parkinson's prevalence.

The following table provides an overview of the study-level and country covariates used in the Parkinson's model.

Covariate	Measure	Beta	Exponentiated
Socio-demographic Index	prevalence	1.44<br>(1.26–1.63)	4.22<br>(3.53–5.08)
All MarketScan, year 2012	prevalence	-0.0043<br>(-0.016 to -0.00051)	1.00<br>(0.98–1.00)
All MarketScan, year 2010	prevalence	-0.0083<br>(-0.025 to -0.00021)	0.99<br>(0.97–1.00)
All MarketScan, year 2000	prevalence	-0.0086<br>(-0.022 to -0.00044)	0.99<br>(0.98–1.00)
(Un)Filled diagnostic criteria	prevalence	0.20<br>(0.13–0.27)	1.22<br>(1.14–1.31)

Appendix 3

Parkinson's Disease (Fatal)

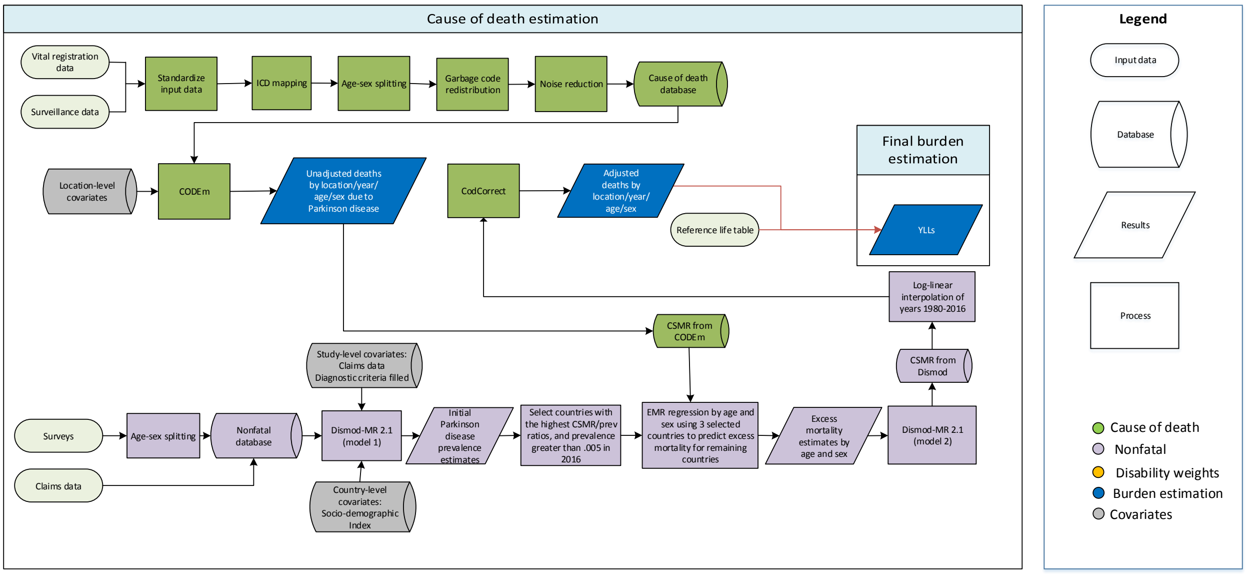

Input Data

In GBD 2016, data used to estimate deaths due to Parkinson's disease included mortality data from vital registration systems and prevalence data from surveys and claims sources.

To add new data for GBD 2016, an updated systematic review was conducted from January 2011 to December 2015, and search terms[3] were set to capture studies for Parkinson's disease. The search yielded 1,433 initial hits and 17 were marked for extraction. Inclusion criteria comprised studies that reported prevalence, incidence, remission rate, excess mortality rate, relative risk of mortality, standardised mortality ratio, or with-condition mortality rate. Studies with no clearly defined sample or that drew from specific clinic/patient organisations were excluded.

Modelling Strategy

Overview

Parkinson's disease mortality rates have more than doubled since 1980 in high-quality vital registration systems such as in the US, Canada, Australia, France, Germany, the United Kingdom, and Finland, while other European countries like the Netherlands, Sweden, and Norway have not seen such increases over time. We have not seen an equivalent increase in prevalence and incidence data sources. Additionally, the greater than 15-fold variation in mortality rates of Parkinson's disease between countries is much greater than the three-fold difference in prevalence and incidence between high-income countries. As it is unlikely that case fatality from Parkinson's disease has dramatically increased over the time period and that it would differ by a very large margin between countries, the

[3] (((Parkinson disease AND epidemiology) AND ("2011/01/01"[PDat]: "2015/12/31"[PDat])) AND (Parkinson disease AND epidemiology))

hypothesis is that certifying and coding practices have changed over time and at a different pace between countries. Therefore, for GBD 2016 we decided to employ a modelling strategy which we have previously used to model mortality from Alzheimer's disease and other dementias. This modelling process avoids spurious large trends over time in the fatal component of the burden of dementia by making dementia mortality rates consistent with the rates observed in 2016 relative to prevalence in countries that are most likely to certify or code Parkinson's disease as an underlying cause of death.

Modelling steps

First, we ran a CODEm model for Parkinson's disease and extracted the mortality rates by age, sex, and geography for 2016. The covariates used are listed below.

Level	Covariate	Direction
1	Cumulative cigarettes (10 years)	0
	Cumulative cigarettes (5 years)	0
2	Absolute latitude	+
	Proportion with access to improved sanitation	0
	Proportion with access to improved water source	0
	Mean serum total cholesterol (mmol/L)	+
	Fruit consumption (grams/day energy adjusted)	-
	Healthcare access and quality index	-
3	Socio-demographic index	+
	Lag-distributed income (I$ per capita)	0
	Education (years per capita)	-

Second, we ran a DisMod-MR 2.1 model with all data on incidence, prevalence, and mortality risk (RR, SMR, or with-condition mortality rates) and a setting of zero remission and extracted 2016 prevalence by age, sex, and geography. To account for potential systematic differences between claims and survey data, we crosswalked for each year of claims data.

Third, we selected the three countries (United States, Finland, and Austria) with the highest cause-specific mortality rate (from step 1) to prevalence (from step 2) ratio in 2016, which also had an age-standardised prevalence rate greater than 0.0005 and a population greater than 1 million.

Fourth, we used a linear effects regression with dummies on age group and sex to predict excess mortality (EMR; ie, the ratio of cause-specific mortality rate and prevalence) by age and sex, the results of which are found in the tables below.

Fifth, these estimates were added to a second DisMod-MR 2.1 model as pertaining to the full 1990–2016 estimation period. For the three countries included in the regression, we retained their age- and sex-specific ratios and entered those also as pertaining to the full 1990–2016 estimation period.

Sixth, we took the predictions of cause-specific mortality by age, sex, geography, and year that DisMod-MR 2.1 calculated as being consistent with the data on incidence, prevalence, and the priors on excess mortality from step five. Socio-demographic Index was used as a country-level covariate. We excluded data for standardised mortality ratio, with-condition mortality rate, and relative risk as we wanted to estimate cause-specific mortality rates that were consistent with the level of excess mortality from the three chosen countries in 2016.

Seventh, because DisMod-MR 2.1 only produces estimates in five-year intervals, we expanded the time series by log-linear interpolation. Values for 1980–1990 were generated using a regression on the entire time series with Socio-demographic Index included as a predictor.

Lastly, before adding the Parkinson's mortality estimates into CodCorrect, we proportionately retrieved the difference in deaths between those estimated in CODEm and those estimated in step 7 from a set of "target causes" which were identified as causes of death in cohort studies of persons with dementia. We assumed the same target causes for dementia would apply to Parkinson's disease as well. The target causes included lower respiratory infections, protein-energy malnutrition, other nutritional deficiencies, cerebrovascular disease, interstitial nephritis and urinary tract infections, decubitus ulcer, and pulmonary aspiration and foreign body in airway.[4] [5] [6] [7] More information on this process is located in the 2016 GBD cause of death capstone paper.[8]

**Fixed effect coefficients of EMR regression. Outcome: ln(EMR)**					
**Independent variables**	**Coef**	**Std. error**	**P value**	**95% confidence interval**	
Male	0.214	0.074	0.006	0.069	0.359
Age 40–59	-3.522	0.157	0.000	-3.829	-3.214
Age 60–64	-2.716	0.157	0.000	-3.024	-2.409
Age 65–69	-2.236	0.157	0.000	-2.544	-1.929
Age 70–74	-1.686	0.157	0.000	-1.993	-1.378
Age 75–80	-1.194	0.157	0.000	-1.502	-0.887
Age 80–84	-0.779	0.157	0.000	-1.087	-0.471
Age 85–89	-0.493	0.157	0.003	-0.800	-0.185
Age 90–94	-0.203	0.157	0.202	-0.511	0.104
Constant	-2.097	0.117	0.000	-2.326	-1.867

**Predicted EMR values by age and sex (95% CI)**		
	**Male**	**Female**
Age 40–59	0.005 (0.004–0.006)	0.004 (0.003–0.005)
Age 60–64	0.01 (0.008–0.013)	0.008 (0.006–0.01)
Age 65–69	0.016 (0.013–0.02)	0.013 (0.01–0.016)
Age 70–74	0.028 (0.023–0.035)	0.023 (0.018–0.029)
Age 75–80	0.047 (0.037–0.059)	0.037 (0.029–0.046)
Age 80–84	0.071 (0.055–0.089)	0.057 (0.045–0.07)
Age 85–89	0.093 (0.073–0.117)	0.076 (0.06–0.093)
Age 90–94	0.126 (0.099–0.155)	0.101 (0.08–0.127)
Age 95+	0.154 (0.122–0.191)	0.123 (0.097–0.153)

[4] Brunnström HR, Englund EM. Cause of death in patients with dementia disorders. *European journal of neurology*. 2009 Apr 1;16(4):488-92.
[5] Thomas BM, Starr JM, Whalley LJ. Death certification in treated cases of presenile Alzheimer's disease and vascular dementia in Scotland. *Age and Ageing.* 1997 Sep 1;26(5):401-6.
[6] Todd S, Barr S, Roberts M, Passmore AP. Survival in dementia and predictors of mortality: a review. *International journal of geriatric psychiatry*. 2013 Nov 1;28(11):1109-24.
[7] Keene J, Hope T, Fairburn CG, Jacoby R. Death and dementia. *International journal of geriatric psychiatry.* 2001 Oct 1;16(10):969-74.
[8] GBD 2016 Causes of Death Collaborators. Global, regional, and national age-sex specific mortality for 264 causes of death, 1980-2016: a systematic analysis for the Global Burden of Disease Study 2016. Lancet 2017; 390: 1151–210.

Appendix 4

Count of data sources used in nonfatal modeling for Parkinson's disease by 21 regions in 2016

region_name	incidence	prevalence	remission	mortality	hospital_claims
East Asia	3	9	0	0	0
Southeast Asia	0	1	0	0	0
Oceania	0	1	0	0	0
Central Asia	0	0	0	0	0
Central Europe	1	1	0	0	0
Eastern Europe	2	1	0	0	0
High-income Asia Pacific	2	7	0	1	0
Australasia	1	4	0	0	0
Western Europe	18	40	0	6	0
Southern Latin America	1	4	0	0	0
High-income North America	5	7	0	4	3
Caribbean	0	1	0	0	0
Andean Latin America	0	1	0	0	0
Central Latin America	0	0	0	0	0
Tropical Latin America	0	0	0	0	0
North Africa and Middle East	0	7	0	0	0
South Asia	1	3	0	0	0
Central Sub-Saharan Africa	0	0	0	0	0
Eastern Sub-Saharan Africa	0	3	0	0	0
Southern Sub-Saharan Africa	0	0	0	0	0
Western Sub-Saharan Africa	0	1	0	0	0
**Total**	**34**	**91**	**0**	**11**	**3**

Appendix 5

GATHER checklist

#	GATHER checklist item	Description of compliance	Reference
**Objectives and funding**			
1	Define the indicators, populations, and time periods for which estimates were made.	Narrative provided in paper and appendix describing indicators, definitions, and populations	Main text (Methods) and appendix
2	List the funding sources for the work.	Funding sources listed in paper	Summary (Funding)
**Data Inputs**			
For all data inputs from multiple sources that are synthesized as part of the study:			
3	Describe how the data were identified and how the data were accessed.	Narrative description of data seeking methods provided	Main text (Methods) and appendix
4	Specify the inclusion and exclusion criteria. Identify all ad-hoc exclusions.	Narrative about inclusion and exclusion criteria by data type provided; Adhoc exclusions in cause specific write ups	Main text (Methods) and appendix
5	Provide information on all included data sources and their main characteristics. For each data source used, report reference information or contact name/institution, population represented, data collection method, year(s) of data collection, sex and age range, diagnostic criteria or measurement method, and sample size, as relevant.	An interactive, online data source tool that provides metadata for data sources by component, geography, cause, risk, or impairment has been developed	Online data citation tools: http://ghdx.healthdata.org/gbd-2016
6	Identify and describe any categories of input data that have potentially important biases (e.g., based on characteristics listed in item 5).	Summary of known biases by cause included in appendix	Appendix
For data inputs that contribute to the analysis but were not synthesized as part of the study:			
7	Describe and give sources for any other data inputs.	Included in online data source tool	http://ghdx.healthdata.org/gbd-2016
For all data inputs:			
8	Provide all data inputs in a file format from which data can be efficiently extracted (e.g., a spreadsheet as opposed to a PDF), including all relevant meta-data listed in item 5. For any data inputs that cannot be shared due to ethical or legal reasons, such as third-party ownership, provide a contact name or the name of the institution that retains the right to the data.	Downloads of input data available through online tools, including data visualization tools and data query tools; input data not available in tools will be made available upon request	Online data visualization tools, data query tools, and the Global Health Data Exchange
**Data analysis**			
9	Provide a conceptual overview of the data analysis method. A diagram may be helpful.	Flow diagrams of the overall methodological processes, as well as	Main text (Methods) and appendix

		cause-specific modelling processes, have been provided	
10	Provide a detailed description of all steps of the analysis, including mathematical formulae. This description should cover, as relevant, data cleaning, data pre-processing, data adjustments and weighting of data sources, and mathematical or statistical model(s).	Flow diagrams and corresponding methodological write-ups for each cause, as well as the databases and modelling processes, have been provided	Main text (Methods) and appendix
11	Describe how candidate models were evaluated and how the final model(s) were selected.	Provided in the methodological write-ups	Appendix
12	Provide the results of an evaluation of model performance, if done, as well as the results of any relevant sensitivity analysis.	Provided in the methodological write-ups	Appendix
13	Describe methods for calculating uncertainty of the estimates. State which sources of uncertainty were, and were not, accounted for in the uncertainty analysis.	Appendix	Appendix
14	State how analytic or statistical source code used to generate estimates can be accessed.	Appendix	http://ghdx.healthdata.org/gbd-2016-code
**Results and Discussion**			
15	Provide published estimates in a file format from which data can be efficiently extracted.	GBD 2016 results are available through online data visualization tools, the Global Health Data Exchange, and the online data query tool	Main text, and online data tools (data visualization tools, data query tools, and the Global Health Data Exchange)
16	Report a quantitative measure of the uncertainty of the estimates (e.g. uncertainty intervals).	Uncertainty intervals are provided with all results	Main text, appendix, and online data tools (data visualization tools, data query tools, and the Global Health Data Exchange)
17	Interpret results in light of existing evidence. If updating a previous set of estimates, describe the reasons for changes in estimates.	Discussion of methodological changes between GBD rounds provided in the narrative of the manuscript and appendix	Main text (Methods and Discussion) and appendix
18	Discuss limitations of the estimates. Include a discussion of any modelling assumptions or data limitations that affect interpretation of the estimates.	Discussion of limitations provided in the narrative of the main paper, as well as in the methodological write-ups in the appendix	Main text (Limitations) and appendix
